# COVID-19 and resilience of healthcare systems in ten countries

**DOI:** 10.1038/s41591-022-01750-1

**Published:** 2022-03-14

**Authors:** Catherine Arsenault, Anna Gage, Min Kyung Kim, Neena R. Kapoor, Patricia Akweongo, Freddie Amponsah, Amit Aryal, Daisuke Asai, John Koku Awoonor-Williams, Wondimu Ayele, Paula Bedregal, Svetlana V. Doubova, Mahesh Dulal, Dominic Dormenyo Gadeka, Georgiana Gordon-Strachan, Damen Haile Mariam, Dilipkumar Hensman, Jean Paul Joseph, Phanuwich Kaewkamjornchai, Munir Kassa Eshetu, Solomon Kassahun Gelaw, Shogo Kubota, Borwornsom Leerapan, Paula Margozzini, Anagaw Derseh Mebratie, Suresh Mehata, Mosa Moshabela, Londiwe Mthethwa, Adiam Nega, Juhwan Oh, Sookyung Park, Álvaro Passi-Solar, Ricardo Pérez-Cuevas, Alongkhone Phengsavanh, Tarylee Reddy, Thanitsara Rittiphairoj, Jaime C. Sapag, Roody Thermidor, Boikhutso Tlou, Francisco Valenzuela Guiñez, Sebastian Bauhoff, Margaret E. Kruk

**Affiliations:** 1 Department of Global Health and Population, Harvard T.H. Chan School of Public Health, Boston MA, USA; 2 Seoul National University College of Medicine, Seoul, South Korea; 3 School of Public Health, University of Ghana, Accra, Ghana; 4 Policy, Planning, Monitoring and Evaluation, Ghana Health Services, Accra, Ghana; 5 Office of the Member of Federal Parliament Gagan Kumar Thapa, Kathmandu, Nepal; 6 World Health Organization, Vientiane, Lao People’s Democratic Republic, Vientiane, Laos; 7 School of Public Health, Addis Ababa University, Addis Ababa, Ethiopia; 8 Public Health Department, Faculty of Medicine, Pontificia Universidad Católica de Chile, Santiago, Chile; 9 Epidemiology and Health Services Research Unit CMN Siglo XXI, Mexican Institute of Social Security, Mexico City, Mexico; 10 Caribbean Institute for Health Research, University of West Indies, Kingston, Jamaica; 11 Hôpital Universitaire de Mirebalais, Zanmi Lasante, Arrondissement de Mirebalais, Mirebalais, Haïti; 12 Faculty of Medicine Ramathibodi Hospital, Madidol University, Bangkok, Thailand; 13 Ministry of Health of Ethiopia, Addis Ababa, Ethiopia; 14 Ministry of Health and Population, Government of Nepal, Kathmandu, Nepal; 15 School of Nursing and Public Health, University of KwaZulu-Natal, Durban, South Africa; 16 Korea National Health Insurance Services, Health Insurance Research Institute, Gangwon-do, South Korea; 17 Division of Social Protection and Health, Inter-American Development Bank, Kingston, Jamaica; 18 Faculty of Medicine, University of Health Sciences, Vientiane, Lao People’s Democratic Republic, Vientiane, Laos; 19 Biostatistics Unit, South African Medical Research Council, Durban, South Africa; 20 Studies and Planning Unit, Ministry of Public Health and Population, Port-au-Prince, Haiti

**Keywords:** Health services, Health policy, Developing world

## Abstract

Declines in health service use during the Coronavirus Disease 2019 (COVID-19) pandemic could have important effects on population health. In this study, we used an interrupted time series design to assess the immediate effect of the pandemic on 31 health services in two low-income (Ethiopia and Haiti), six middle-income﻿ (Ghana, Lao People’s Democratic Republic, Mexico, Nepal, South Africa and Thailand) and high-income (Chile and South Korea) countries. Despite efforts to maintain health services, disruptions of varying magnitude and duration were found in every country, with no clear patterns by country income group or pandemic intensity. Disruptions in health services often preceded COVID-19 waves. Cancer screenings, TB screening and detection and HIV testing were most affected (26–96% declines). Total outpatient visits declined by 9–40% at national levels and remained lower than predicted by the end of 2020. Maternal health services were disrupted in approximately half of the countries, with declines ranging from 5% to 33%. Child vaccinations were disrupted for shorter periods, but we estimate that catch-up campaigns might not have reached all children missed. By contrast, provision of antiretrovirals for HIV was not affected. By the end of 2020, substantial disruptions remained in half of the countries. Preliminary data for 2021 indicate that disruptions likely persisted. Although a portion of the declines observed might result from decreased needs during lockdowns (from fewer infectious illnesses or injuries), a larger share likely reflects a shortfall of health system resilience. Countries must plan to compensate for missed healthcare during the current pandemic and invest in strategies for better health system resilience for future emergencies.

## Abstract (web summary)

An interrupted time series analysis of 31 healthcare services in ten low-income, middle-income and high-income countries demonstrates that the COVID-19 pandemic caused immediate, heterogeneous and prolonged disruptions in service delivery, highlighting the need for health system resilience in pandemic preparedness.

## Main

During a health crisis such as the COVID-19 pandemic, high-quality and resilient health systems have two tasks: respond to the crisis and maintain provision of other essential health services^1–3^. As of December 2021, more than 270 million people have been infected with COVID-19, leading to 5.3 million deaths globally^4^. Although effective vaccines are now available, their distribution remains widely inequitable, and new waves of cases and variants continue to emerge^5^.

Owing to its prolonged nature, the pandemic will have widespread indirect effects. Among these effects, concern is increasing that lasting declines in healthcare use could reverse decades of progress in improving health outcomes in low- and middle-income countries (LMICs) and puts people at increased risk of avoidable illness and death. Several reasons have been cited for declining healthcare use during the pandemic, including the public’s fear of becoming infected while visiting health facilities, the suspension or cancellation of non-COVID-19 care as well as barriers imposed by lockdown policies (for example, curfews, transport closures and stay-at-home orders)^6,7^. Although some services can potentially be delayed during an emergency, other, more urgent, services cannot. Several modeling studies point to potentially substantial negative health effects from a reduction in essential healthcare^8–10^. Several studies have described the magnitude of decline in healthcare use during COVID-19, but these have generally been limited in scale and scope^11–16^. Many studies have covered only the first few months of the pandemic, small geographic areas or a limited number of health facilities and types of care. Most published studies also report on disruptions in high-income countries^17,18^.

Understanding how a broader range of health services have been affected across various contexts is essential for future health system planning and pandemic preparedness. In April 2020, the QuEST network, an initiative focused on measuring and improving health system quality through multi-country partnerships, undertook a study to monitor health system resilience during the pandemic using data from national health information systems^19^. The present study is the culmination of that effort. We assessed the effects of the pandemic on 31 health services in ten countries, including two low-income countries (Ethiopia and Haiti), three lower-middle-income countries (Ghana, Lao People’s Democratic Republic and Nepal), three upper-middle-income countries (Mexico, South Africa and Thailand) and two high-income countries (Chile and South Korea). These countries vary in health expenditures, health system structures, COVID-19 severity and their national response to the pandemic. We focused on the immediate effects of the pandemic in these countries and describe trends in service resumption. We also estimate the magnitude of missed healthcare appointments per 1,000 population and discuss country-specific factors that have promoted resilience or worsened disruptions.

## Results

### Data and health services analyzed

**Fig. 1:**
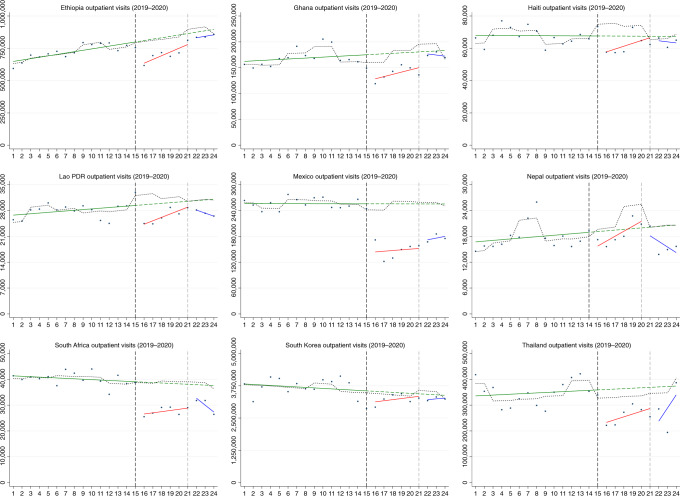
Trends in total outpatient visits in nine countries from January 2019 to December 2020. The blue dots are the average monthly number of outpatient visits per sub-national unit (observed). The *x* axes are months 1–24, representing January 2019 to December 2020, except in Nepal where they correspond to 15 January 2019 to 13 January 2021. The *y* axes are the total services provided. The vertical black line shows the beginning of the COVID-19 pandemic, and the vertical gray line shows the beginning of the potential resumption period (last quarter of 2020). The green trend line is the predicted trend based on pre-COVID-19 months. The black dotted trend line is the temporal trend adjusted for seasonality. The red line is the temporal trend in the 6 months after the pandemic was declared (April to September 2020). The blue line is the temporal trend in the last quarter of 2020. Trends extended to June 2021 are available for seven countries in Extended Data Fig. 1. In South Africa, outpatient visits are reported only by hospitals and, thus, represent only a fraction of service provision. In Chile, outpatient visits were not available.

We modeled the pandemic’s impact using an interrupted time series (ITS) design (Methods)^20^. We used administrative and Routine Health Information System (RHIS) data on the number of health services provided each month for the period of January 2019 to December 2020. These datasets covered varying health sectors or sub-national regions and represent the services provided by a total of 94,655 health facilities in ten countries (Table 1). In South Africa, data were obtained from the KwaZulu-Natal province only, and we used these data as a proxy for the country. The interruption was defined as the declaration of a pandemic by the World Health Organization on 11 March 2020, resulting in 15 months of baseline and 9 months of follow-up during the COVID-19 pandemic^21^. Trends in services until June 2021 are also available for a subset of indicators and countries. The primary parameters of interest were (1) the change in the level of health services after the declaration of the pandemic and (2) the remaining difference in level at the end of 2020 (to assess whether services had resumed to pre-pandemic levels). The two parameters are reflected in Fig. 1 by the red and blue trend lines, respectively. We express results in relative terms as the percent change from the average in the pre-COVID-19 period.

**Table 1:** Description of the datasets

Country	Name of the routine health information system or administrative dataset	Dataset coverage	Total number of facilities reporting to the health information system	Number and type of sub-national units for modeling
**Chile**	Sistemas de Información del Ministerio de Salud	Public sector (serves approximately 80% of the population) and private sector for some indicators^a^	2,821 (ref. ^67^)	16 regions
**Ethiopia**	Health Management Information System (DHIS2 platform), Ministry of Health	All health facilities in the country (except Tigray region)^b^	24,481 (ref. ^68^)	10 regions
**Ghana**	District Health Information Management System (DHIMS) (DHIS2 platform), Ghana Health Services	All health facilities in the country	7,060 (ref. ^69^)	16 regions
**Haiti**	Système d’Information Sanitaire Unique (SISNU) (DHIS2 platform), Ministère de la Santé Publique et de la Population	All health facilities in the country	1,033 (ref. ^70^)	10 departments
**Lao PDR**	Health Management Information System (DHIS2 platform), Ministry of Health	Public health facilities only (the public system is the predominant provider of care)^c^	1,277 (ref. ^71^)	18 provinces
**Mexico**	Sistema de información del Instituto Mexicano del Seguro Social (IMSS)	IMSS (serves approximately half the population)	1,830 (ref. ^72^)	35 delegations
**Nepal**	Health Management Information System (DHIS2 platform), Ministry of Health and Population, Department of Health Services	All health facilities in the country	7,605 (ref. ^73^)	77 districts
**South Africa**	Health Management Information System (DHIS2 platform), National Department of Health	All health facilities in the KwaZulu-Natal province only	769 (ref. ^74^)	11 metropolitan and district municipalities
**South Korea**	National Health Insurance Service (NHIS) Health Facility Claims Database	All health facilities in the country	36,125 (ref. ^75^)	17 provinces
**Thailand**	National Health Database of the Ministry of Public Health (43-folders dataset)	Public health facilities only (serves approximately 70% of the population)	11,654 (ref. ^76^)	77 provinces

^a^In Chile, deliveries, C-sections, inpatient admissions, discharges for child pneumonia and road traffic accidents include data from both public and private hospitals.

^b^Reporting in Tigray was suspended starting October 2020 due to political instability. The Tigray region was excluded from the analysis.

^c^There is no official estimate for the share of the private sector in Laos, which is loosely regulated. Healthcare is predominantly delivered by government-owned facilities. For example, private sector counts for only 2.6% of all facility births, according to the latest Multiple Indicator Cluster Survey.

We analyzed a total of 31 health services for a broad range of health needs, including reproductive, maternal and child health services; child vaccinations; services for HIV, TB, malaria and chronic diseases; and road traffic accidents. Summative measures of health system contacts were also included (for example, total number of outpatient visits and inpatient admissions). Detailed definitions are in Supplementary Table 1.

### Immediate effect after the declaration of the pandemic

**Fig. 2:**
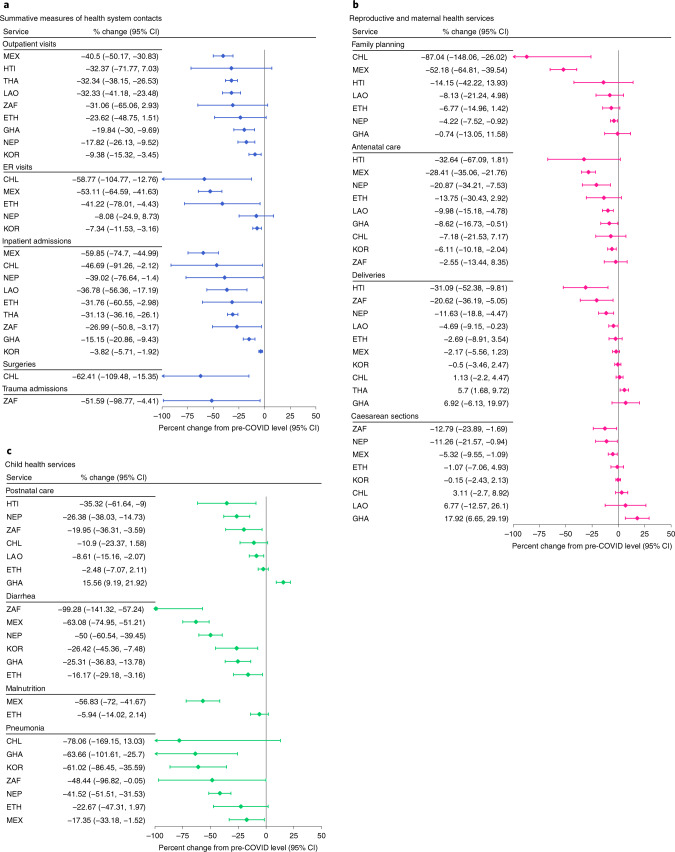
Immediate effect of the pandemic on summative measures and reproductive, maternal and child health services. Effect estimates are expressed as the percent change in service level after the declaration of the pandemic compared to the average level pre-COVID-19. The percent change from the average pre-COVID-19 is calculated by dividing the coefficient for the COVID-19 period (β2) from regression models to the monthly average in the 15 months pre-COVID-19. Lower and upper limits of the 95% confidence interval (CI) are also divided by the monthly average pre-COVID-19 to be expressed as percentages. Regression coefficients and CIs are in Supplementary Tables 10–19. Countries are represented with International Organization for Standardization country codes.

**Fig. 3:**
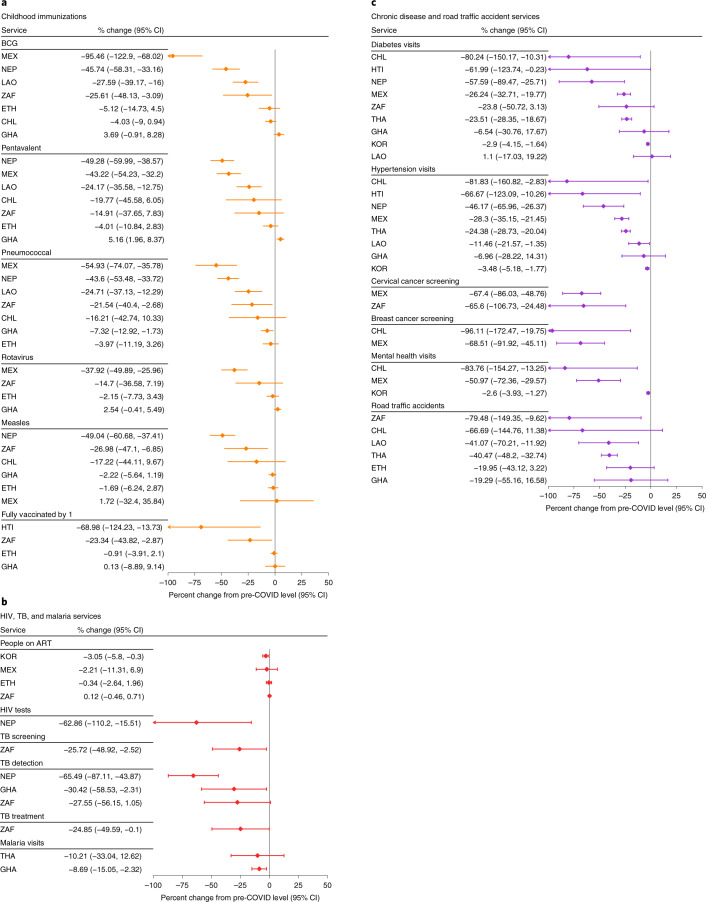
Immediate effect of the pandemic on childhood immunizations and services for HIV, TB, malaria, chronic diseases and road traffic accidents. Effect estimates are expressed as the percent change in service level after the declaration of the pandemic compared to the average level pre-COVID-19. The percent change from the average pre-COVID-19 is calculated by dividing the coefficient for the COVID-19 period (β2) from regression models to the monthly average in the 15 months pre-COVID-19. Lower and upper limits of the 95% confidence interval (CI) are also divided by the monthly average pre-COVID-19 to be expressed as percentages. Regression coefficients and CIs are in Supplementary Tables 10–19. Childhood immunizations are for the number of children who received the final dose for the pentavalent vaccine, the pneumococcal conjugate vaccine and the rotavirus vaccine. Measles vaccination is for the number of children who received the first dose in Ethiopia and Nepal, the second dose in Mexico and South Africa and both first and second doses in Ghana. Full vaccination by age 1 is according to the national immunization schedule. Countries are represented with International Organization for Standardization country codes.

The forest plots in Figs. 2 and 3 show the immediate effect of the pandemic on health service levels. The types and number of health services analyzed varied by country. Nonetheless, no country in this analysis was spared from disruptions. The five summative measures of health system contacts—outpatient visits, emergency room visits, inpatient admissions, surgeries and trauma admissions—declined in all countries reporting these indicators (Fig. 2a). Reproductive and maternal health services were overall less affected than the summative measures (Fig. 2b). Family planning visits declined by more than 10% in three of seven countries: Chile, Haiti and Mexico. Deliveries and C-sections declined by 5–31% in five countries but were stable elsewhere (Fig. 2b). The number of children younger than 5 years of age seeking treatment for diarrhea or pneumonia declined by 16–99% in all seven countries reporting (Fig. 2c). Declines in childhood immunizations greater than 10% were also observed in all countries reporting, except in Ethiopia and Ghana (Fig. 3a). However, not all vaccines were similarly affected within countries. For example, Mexico had a 95% decline in Bacille Calmette-Guérin (BCG) vaccination but a 2% increase in measles vaccination. TB services (screening, detection or treatment initiation) were reported by Ghana, Nepal and South Africa and declined by 25–65% in these three countries (Fig. 3b). Malaria visits declined by 9% and 10% in Ghana and Thailand, respectively. The provision of antiretroviral therapy (ART) was the most resilient health service assessed, with virtually no change across the four countries reporting: Ethiopia, Mexico, South Africa and South Korea (Fig. 3b). By contrast, the number of people tested for HIV declined by 63% in Nepal. Consultations for diabetes or hypertension declined by more than 20% in six countries: Chile, Haiti, Mexico, Nepal, South Africa and Thailand (Fig. 3c). Screening for breast cancer declined by 69% in Mexico and by 96% in Chile and was one of the services most affected by the pandemic (Fig. 3c). Cervical cancer screening declined by 67% in Mexico and 66% in South Africa (Fig. 3c). Mental health services declined by 3% in South Korea and by 51% and 84% in Mexico and Chile, respectively (Fig. 3c). Road traffic accidents also declined by 19–79% in all five countries reporting, likely influenced by reductions in mobility rather than care-seeking (Fig. 3c)^22^. By contrast, we found statistically significant increases in three services in Ghana (C-sections, postnatal care and pentavalent vaccinations). Efforts to maintain continuity of reproductive, maternal, newborn and child health services in Ghana may have led to an increase in service use during that period^23^.

### Resumption in services

**Fig. 4:**
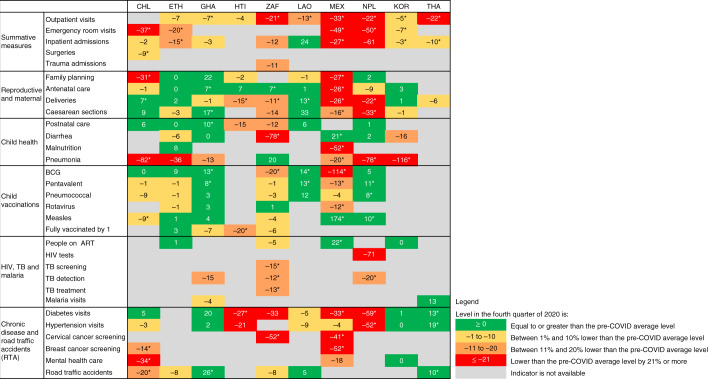
Resumption in services by the fourth quarter of 2020. Estimates in the cells represent the remaining level change compared to the average level pre-COVID-19 and are expressed as the percent difference from the average pre-COVID-19. The percent difference in quarter 4 of 2020 to the average pre-COVID-19 is calculated by dividing the coefficient for the quarter 4 period (β_4_) from regression models to the monthly average in the 15 months pre-COVID-19. Asterisks (*) indicate statistical significance (*P* < 0.05). Countries are represented with International Organization for Standardization country codes.

**Extended Data Fig. 1:**
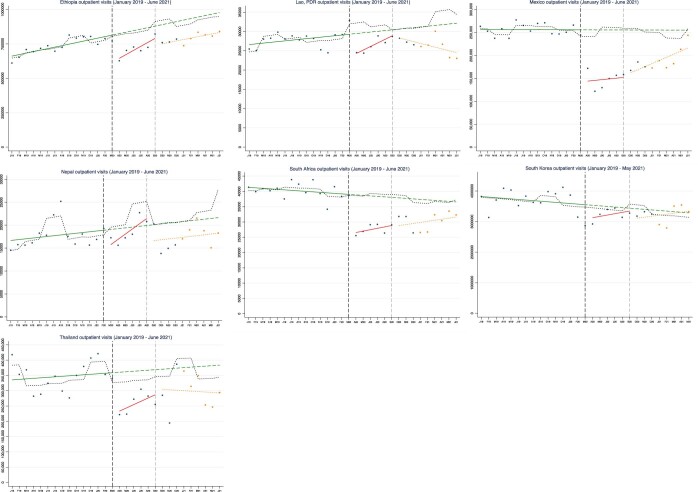
Trends in total outpatient visits in seven countries from January 2019 to June 2021. The dots are the average monthly number of outpatient visits per sub-national unit (observed) in 2019-2020 (blue) and 2021 (orange). The x-axis is January 2019 to May 2021 in Korea and January 2019 to June 2021 in Ethiopia, Laos, Mexico, Thailand, and South Africa. In Nepal, the last month ends on July 15, 2021, due to a different calendar. The y-axes are the total services provided. The vertical black line shows the beginning of the COVID-19 pandemic and the vertical grey line, the beginning of the potential resumption period (last quarter of 2020). The green trend line is the predicted trend based on pre-COVID-19 months. The black dotted line is the temporal trend adjusted for seasonality. The red line is the temporal trend in the 6 months after the pandemic was declared (April to September 2020). The orange dotted line is the trend from October 2020 to May or June 2021. In Ghana and Haiti, these data were only available until December 2020 and are presented in the main text. In South Africa outpatient visits are only reported by hospitals and thus only represent a fraction of service provision. In Chile, outpatient visits were not available.

**Extended Data Fig. 2:**
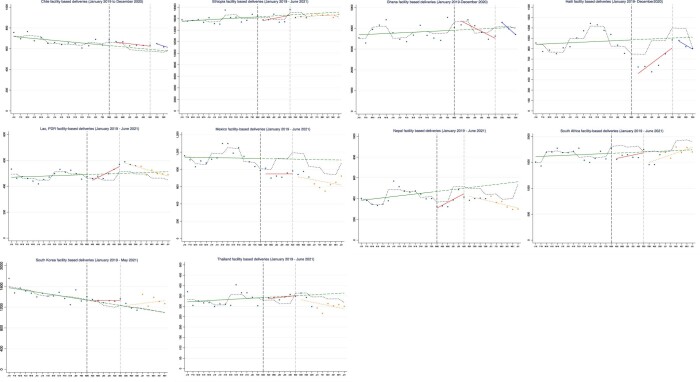
Trends in facility-based deliveries in ten countries from January 2019 to June 2021. The dots are the average monthly number of deliveries per sub-national unit (observed) in 2019-2020 (blue) and 2021 (orange). The x-axis is January 2019 to December 2020 in Chile, Ghana and Haiti, January 2019 to May 2021 in Korea, and January 2019 to June 2021 in Ethiopia, Laos, Mexico, South Africa, and Thailand. In Nepal, the last month ends on July 15, 2021, due to a different calendar. The y-axes are the total services provided. The vertical black line shows the beginning of the COVID-19 pandemic and the vertical grey line, the beginning of the potential resumption period (starting October 2020). The green trend line is the predicted trend based on pre-COVID-19 months. The black dotted line is the temporal trend adjusted for seasonality. The red line is the temporal trend in the 6 months after the pandemic was declared (April to September 2020). The blue line is the temporal trend in the last quarter of 2020 (in countries where 2021 data were not available). The orange dotted line is the trend from October 2020 to May or June 2021.

**Extended Data Fig. 3:**
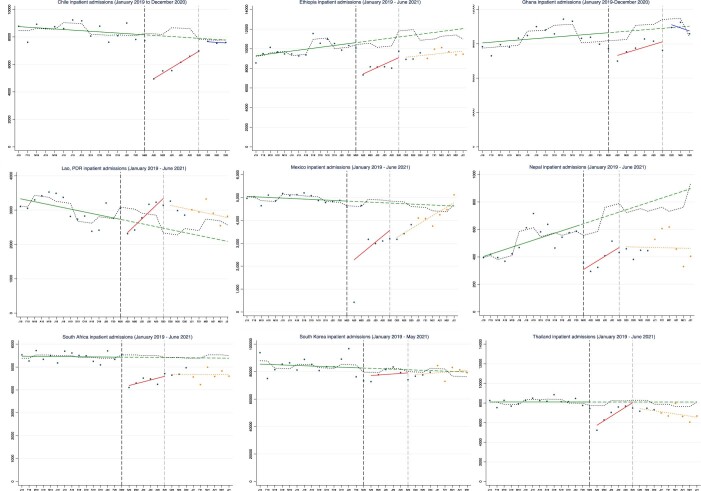
Trends in inpatient admissions in nine countries from January 2019 to June 2021. The dots are the average monthly number of inpatient admissions per sub-national unit (observed) in 2019-2020 (blue) and 2021 (orange). The x-axis is January 2019 to December 2020 in Chile and Ghana, January 2019 to May 2021 in Korea and January 2019 to June 2021 in Ethiopia, Laos, Mexico, South Africa, and Thailand. In Nepal, the last month ends on July 15, 2021, due to a different calendar. The y-axes are the total services provided. The vertical black line shows the beginning of the COVID-19 pandemic and the vertical grey line, the beginning of the potential resumption period (starting October 2020). The green trend line is the predicted trend based on pre-COVID-19 months. The black dotted line is the temporal trend adjusted for seasonality. The red line is the temporal trend in the 6 months after the pandemic was declared (April to September 2020). The blue line is the temporal trend in the last quarter of 2020 (in countries where 2021 data were not available). The orange dotted line is the trend from October 2020 to May or June 2021.

**Extended Data Fig. 4:**
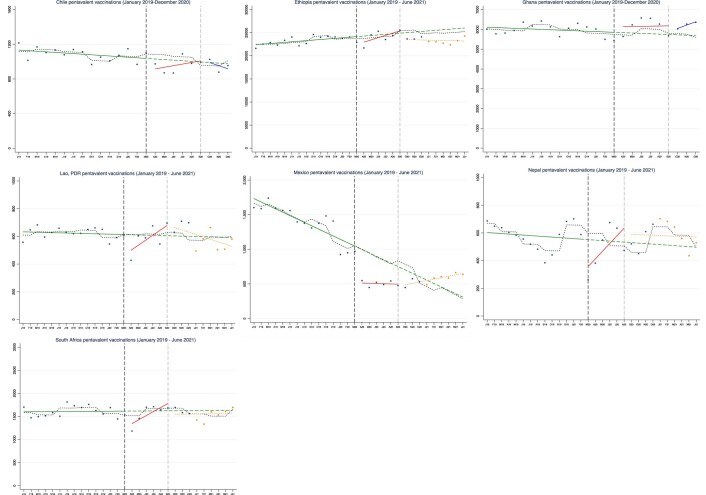
Trends in pentavalent vaccinations in seven countries from January 2019 to June 2021. The dots are the average monthly number of pentavalent vaccinations per sub-national unit (observed) in 2019-2020 (blue) and 2021 (orange). The x-axis is January 2019 to December 2020 in Chile and Ghana, and January 2019 to June 2021 in Ethiopia, Laos, Mexico and South Africa. In Nepal, the last month ends on July 15, 2021, due to a different calendar. The y-axes are the total services provided. The vertical black line shows the beginning of the COVID-19 pandemic and the vertical grey line, the beginning of the potential resumption period (starting October 2020). The green trend line is the predicted trend based on pre-COVID-19 months. The black dotted line is the temporal trend adjusted for seasonality. The red line is the temporal trend in the 6 months after the pandemic was declared (April to September 2020). The blue line is the temporal trend in the last quarter of 2020 (in countries where 2021 data were not available). The orange dotted line is the trend from October 2020 to May or June 2021.

**Extended Data Fig. 5:**
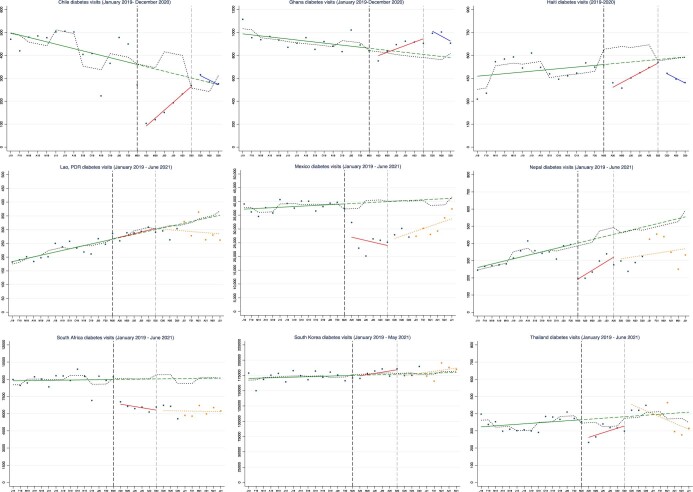
Trends in visits for diabetes in nine countries from January 2019 to June 2021. The dots are the average monthly number of diabetes visits per sub-national unit (observed) in 2019-2020 (blue) and 2021 (orange). The x-axis is January 2019 to December 2020 in Chile, Ghana and Haiti, January 2019 to May 2021 in Korea and January 2019 to June 2021 in Laos, Mexico, South Africa and Thailand. In Nepal, the last month ends on July 15, 2021, due to a different calendar. The y-axes are the total services provided. The vertical black line shows the beginning of the COVID-19 pandemic and the vertical grey line, the beginning of the potential resumption period (starting October 2020). The green trend line is the predicted trend based on pre-COVID-19 months. The black dotted line is the temporal trend adjusted for seasonality. The red line is the temporal trend in the 6 months after the pandemic was declared (April to September 2020). The blue line is the temporal trend in the last quarter of 2020 (in countries where 2021 data were not available). The orange dotted line is the trend from October 2020 to May or June 2021.

Using regression models, we assessed whether services had returned to pre-pandemic predicted levels by the end of 2020 (Methods). Although the COVID-19 pandemic continued beyond 2020 globally, health systems should have adjusted to the continuing circumstances and adapted service delivery. We found that, by the last quarter of 2020, most services had improved compared to the early months of the pandemic, but not all had returned to pre-pandemic levels. Figure 4 shows the percent differences in levels between the end of 2020 and the average pre-COVID-19 for all ten countries. Several services were still affected by the end of the year, including in Chile, Haiti, Mexico, Nepal (which had its first COVID-19 wave peak in late October 2020) and South Africa. In Ethiopia, Ghana, Laos, South Korea and Thailand, only a few services were still affected by the last quarter of 2020, including total outpatient and emergency room visits, which remained lower than predicted in all countries. Data for the first half of 2021 are shown in a subset of countries in Extended Data Fig. 1 for outpatient visits, Extended Data Fig. 2 for institutional deliveries, Extended Data Fig. 3 for inpatient admissions, Extended Data Fig. 4 for pentavalent vaccination and Extended Data Fig. 5 for diabetes visits. Overall, countries and services with disruptions in 2020 continued to experience lower use than expected 15 months after the pandemic was declared, except Mexico, which had resumed outpatient visits, pentavalent vaccinations and inpatient admissions to pre-pandemic levels by June 2021. There was also some indication that healthcare use worsened in the second quarter of 2021 in Laos and Thailand (two countries with few COVID-19 cases in 2020 but larger waves in 2021).

### The burden of missed healthcare

**Fig. 5:**
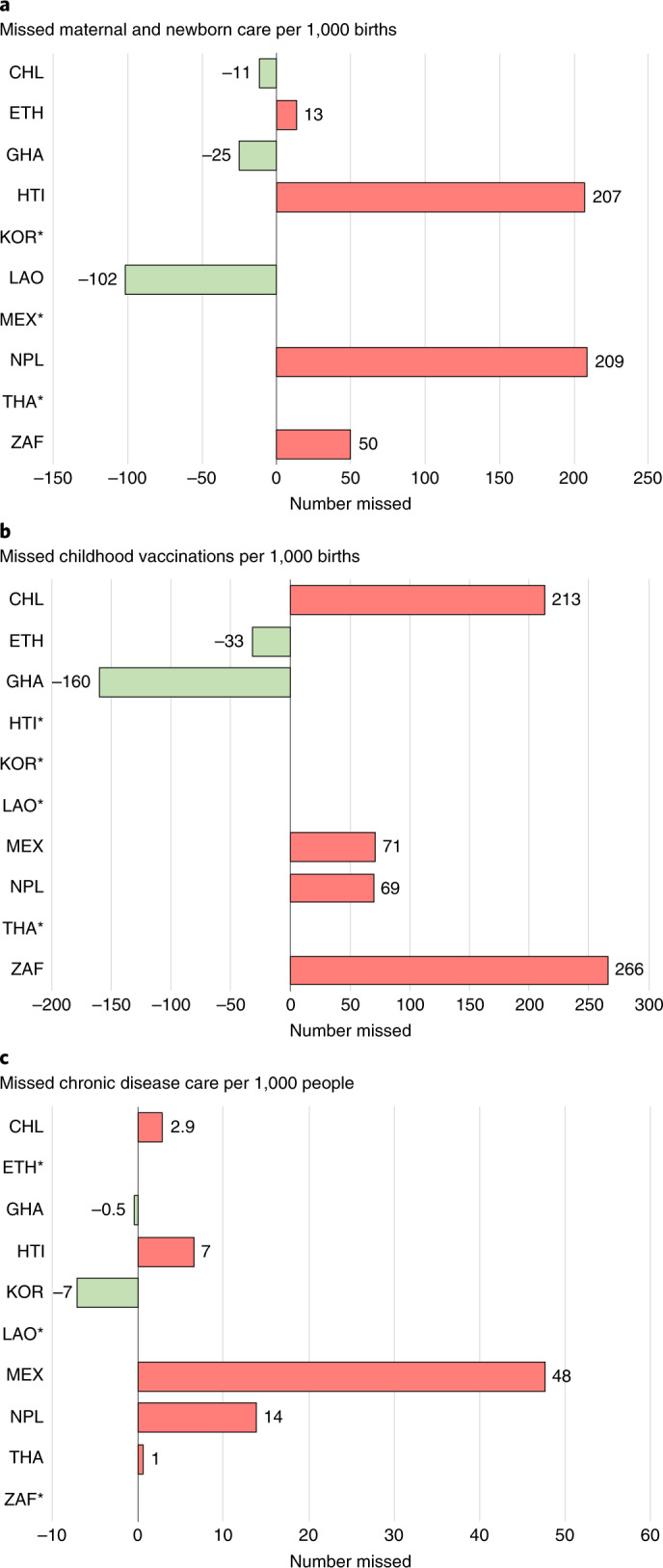
Estimated amount of missed healthcare from April to December 2020. Asterisks (*) indicate that countries were excluded if missing one or more indicator. Missed maternal and newborn care is the sum of antenatal care, delivery and postnatal care. Missed childhood vaccinations is the sum of BCG, pentavalent, pneumococcal and measles vaccinations. Missed chronic disease care is the sum of hypertension and diabetes visits. Negative numbers indicate that more consultations took place than expected according to pre-COVID-19 trend. Annual births are estimated by multiplying the crude birth rate per 1,000 by the population according to most recent population estimates. Countries are represented with International Organization for Standardization country codes.

The cumulative amount of maternal, newborn, child and chronic disease care missed from April to December 2020 is shown in Fig. 5. Haiti and Nepal had the largest estimated amount of missed maternal and newborn care, with 207 and 209 missed visits per 1,000 births, respectively. South Africa had the largest number of missed immunizations, with 266 fewer vaccinations per 1,000 births. Mexico had the largest estimated amount of missed hypertension and diabetes care (48 missed visits per 1,000 people). Throughout the first 9 months of the pandemic, we estimate that a total of 130,431 fewer women gave birth in a health facility across Ghana, Haiti, Mexico, Nepal and South Africa; 131,652 fewer children received their third dose of pentavalent vaccine across Chile, Ethiopia, Mexico, Nepal and South Africa; and 4.6 million fewer people received care for diabetes across Chile, Haiti, Mexico, Nepal, South Africa and Thailand (Supplementary Table 2).

### Factors promoting and disrupting health services

**Extended Data Fig. 6:**
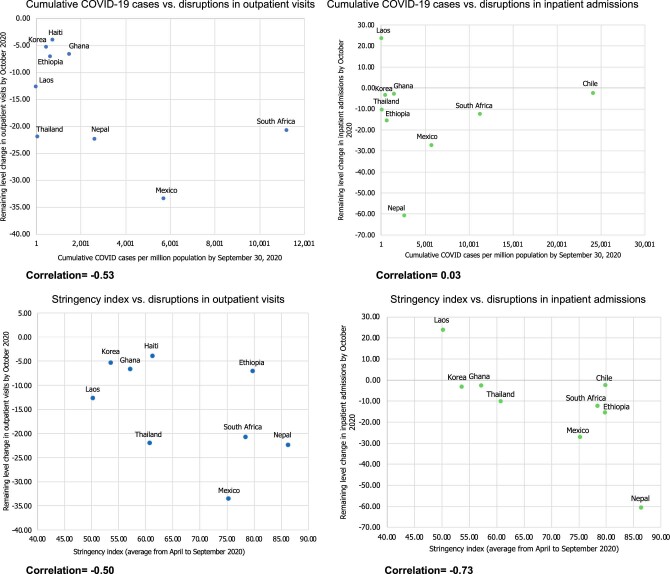
Scatter plots for the association between COVID-19 incidence, the stringency index and health service disruptions. COVID-19 cases are from the Data Repository by the Center for Systems Science and Engineering (CSSE) at Johns Hopkins University. The stringency index was developed by the Oxford Government Response Tracker. The stringency index is a composite measure based on nine response indicators including school closures, workplace closures, cancellation of public events, restrictions on public gatherings, closures on public transport, stay-at-home requirements, public information campaigns, restrictions on internal movements and international travel controls, rescaled to a value from 0 to 100 (100 = strictest).

We conducted a series of sub-analyses to investigate the factors promoting or disrupting health services. First, we investigated whether the stringency of government responses and the cumulative incidence of COVID-19 cases were respectively correlated with the magnitude of disruptions in each country (Extended Data Fig. 6 and Supplementary Table 3). We found moderate correlations between the stringency of policies and the magnitude of disruptions and low to moderate correlations between COVID-19 incidence and health service disruptions. Nonetheless, it is possible that these results mask variations within countries where areas most affected by COVID-19 and lockdowns may have faced worst disruptions. Therefore, we also estimated the immediate effect of the pandemic on health services in the largest metropolitan region in each country and compared those results to national-level disruptions (Supplementary Table 4). We found that service disruptions tended to be larger in metropolitan regions in most countries, except in South Korea.

Finally, working with policymakers and health system stakeholders in each country, we described the strategies and country-specific factors promoting or disrupting health services during the pandemic (Supplementary Table 5). All countries in the study made efforts to maintain essential health service provision during the pandemic, particularly through non-visit care. These included online prescription renewals, telemedicine, digital mental healthcare and home blood pressure monitoring. Other service adaptations promoted service continuity during the pandemic, including scheduling visits to reduce overcrowding, community delivery of drugs for patients with stable chronic conditions, weekend opening hours and special vaccination campaigns or health days. Plans for service prioritization and adaptation during the pandemic were also developed in several countries (Supplementary Table 5).

## Discussion

Using administrative and RHIS data from ten countries, we assessed the effect of the COVID-19 pandemic on a spectrum of health services. We estimated the immediate effect after the declaration of the pandemic on 11 March 2020 and assessed whether services had returned to pre-pandemic levels by the last quarter of 2020. We found declines of varying magnitude and duration in every country. Effects were heterogeneous across countries, and we found no clear patterns in disruptions by country income group or according to the severity of COVID-19 epidemics. The health systems most affected included those in Chile, Haiti, Mexico, Nepal and South Africa. By contrast, Ethiopia and South Korea, which represent the poorest and richest countries, respectively, in our analysis, were among the least affected by health service disruptions.

The magnitude of health service disruptions at national levels also did not appear to be directly driven by COVID-19 severity. Of the ten countries included, six reported fewer than 2,000 cumulative cases per million in 2020 and even fewer deaths (Supplementary Table 3). Only 41 total cases were reported in Laos in 2020. Chile, Mexico, Nepal and South Africa faced higher COVID-19 caseloads, with peaks in June or July (or late October in Nepal). However, health service disruptions were largest in April and May 2020 in all countries, suggesting that they were not caused by overburdened health systems but rather by a combination of policy responses and demand-side factors. Several reasons for reduced healthcare use appeared common across countries: fear of contagion, inability to pay for healthcare due to loss of employment or remuneration, intentional suspension of routine care to leave room for patients with COVID-19, the redeployment of health workers or hospitals to COVID-19 care and prevention and the barriers imposed by COVID-19 lockdowns. Whether the type of COVID-19 response (for example, elimination versus steady-state strategies) or the stringency and length of COVID-19-related lockdowns were associated with the magnitude of disruptions remains unclear and should be investigated further.

On the other hand, we found patterns in disruptions according to the type of health service. Outpatient visits and hospital-based services (including emergency room visits, inpatient admissions, trauma care, accidents and surgeries) declined in every country reporting them, and these disruptions often persisted throughout the period analyzed. Other studies also reported declining inpatient admissions during the COVID-19 pandemic^16,24^. These declines may be explained, in part, by a reduction in need. For example, decreased mobility and bans on alcohol sale in some places have led to fewer accidents and a lower need for trauma care^22,25,26^. Social distancing and mask wearing might have also contributed to reduced spread of infectious diseases. However, the reduction in need is unlikely to account for the entire magnitude of decline. Much of the disruption in tertiary care might reflect that many hospitals were converted into COVID-19 treatment centers and suspended or postponed other services. The prioritization of COVID-19 care also disrupted the availability of intensive care beds, medical supplies and technology for services other than COVID-19. Hospitals also tend to be in urban areas that appear to have been more affected by service disruptions than rural areas (Supplementary Table 4). Declines in emergency room visits may also result from people delaying or foregoing urgent care. For example, studies from France and England suggest that people with chest pains and other symptoms of a myocardial infarction have been reluctant to go to hospitals during the pandemic, leading to a reduction in patients admitted with ST-segment elevation myocardial infarction and an increase in out-of-hospital deaths^27,28^. Persistent disruptions in hospital services could have important consequences, including exacerbating the already high unmet need for surgical care in LMICs^29,30^.

Overall, preventive care, such as routine childhood immunizations, screenings and testing, were among the most affected services. Although some of these services can potentially be delayed for a short time, our estimates indicate that many were not fully restored by the end of 2020. After the declaration of the pandemic, there were declines in child vaccinations of more than 10% in Chile, Haiti, Laos, Mexico, Nepal and South Africa (out of eight countries reporting these data). Several of these countries, in particular Laos and Nepal, were able to resume most vaccinations by the end of the year (Fig. 4). However, our estimates for the number of vaccinations missed from April to December 2020 show that not all vaccinations initially delayed were ultimately given (Fig. 5 and Supplementary Table 2). Observed effects were different across vaccine types. This is likely due to differences in vaccine schedules and delivery modes. For example, BCG is delivered at birth and generally followed the same declines as facility-based childbirth. We reported on five common vaccines (BCG, pentavalent, measles, pneumococcal and rotavirus). Other immunizations with different schedules or distribution modes might have been differently affected, including vaccines against the human papilloma virus, which was likely affected by school closures. Globally, DTP3 and MCV1 vaccination coverage is estimated to have fallen by more than 7% in 2020 compared to expected coverage in the absence of the pandemic^31^. A total of 66 countries also reported postponing at least one vaccination campaign in early 2020, and only 25 reinstated them by the end of the year^32^. These disruptions are expected to lead to future outbreaks of measles and other vaccine-preventable diseases and to an increase in child deaths^8^.

We also found large and persisting declines in breast and cervical cancer screening. Declines in cancer screenings and routine diagnostic work have been reported globally^33–36^. In England, breast cancer diagnostic delays are projected to increase 5-year mortality by 8–10%^35^. Chile and Mexico risk facing similar increases in breast cancer mortality over the next 5 years.

TB case detection declined by 28–66% in Ghana, Nepal and South Africa and remained lower than pre-COVID-19 by the end of 2020. With symptoms similar to COVID-19, such as a cough, fever and breathing difficulties, many people with TB symptoms might have opted to stay home or could have been mistakenly diagnosed with COVID-19 (ref. ^37^). The Global Fund to fight AIDS, TB and Malaria estimates that TB and HIV testing declined by 18–22% in countries supported by the fund. We found even larger declines in HIV testing in Nepal. An increase in untreated TB or HIV could have far-reaching consequences^10,38^. It is unclear whether social distancing may have contributed to reduced TB or HIV transmission. More time spent indoors in crowded households could increase TB transmission.

In contrast, across four countries, we found that the number of people on ART was virtually unaffected during the pandemic. Our findings are consistent with evidence that ART provision was generally maintained during the South African lockdown, whereas HIV testing and ART initiations declined^12,39,40^. Differentiated service delivery (DSD) programs for HIV, where drugs are distributed in decentralized locations, might explain the resilience of ART provision during the pandemic. Unlike traditional care models where visits are frequent and exclusively at the health facility, DSD models entail modifying the location for care (for example, to venues in the community), the frequency of visits (for example, biannually) and the cadre providing the services^41^.

Visits for malaria declined by 9% and 10% in Ghana and Thailand, respectively, but returned to pre-pandemic levels by the end of 2020. These short-term disruptions could still have led to an increase in malaria deaths, particularly if prevention activities (such as bed nets and insecticide spraying) were also disrupted^42,43^.

We found declines in diabetes or hypertension visits of more than 20% in Chile, Haiti, Mexico, Nepal, South Africa and Thailand. Similar disruptions have been reported elsewhere^16,44–46^. Some countries, including Chile, Mexico, South Africa and Thailand, reported implementing strategies to maintain drug adherence during the pandemic for people with these two conditions, such as online refills, community drug delivery or external pick-up points^44,47^. However, it is unclear whether they have been successful in maintaining drug adherence, as our data cover only the number of in-person visits conducted. Hypertension and diabetes management has been a particular challenge for LMIC health systems where the burden of uncontrolled diseases is high^48–50^. The pandemic could prompt policymakers to rethink the frequency of visits required and consider adopting principles of DSD to meet the needs of people living with these conditions^51^. South Africa has adopted such a strategy through the Central Chronic Medicines Dispensing and Distribution program^52^.

We also found large declines in in-person mental health services in Chile and Mexico. Only three countries reported on mental healthcare: Chile, Mexico and South Korea. In Mexico, the indicator was for mental healthcare after an attempted suicide, whereas Chile and South Korea reported on routine mental health consultations. In May 2020, the government of Chile established a digital mental healthcare platform (‘Saludable mente’) to address the rise in mental health disorders during the pandemic. Other countries also integrated mental health interventions, such as telephone hotlines to support frontline health workers and the general population, to their COVID-19 response, including Mexico and South Africa. These programs may have helped mitigate the impact of reduced in-person care, but there is little evidence to date on their effectiveness^53^. The increase in depressive and anxiety disorders reported globally during the pandemic calls for the urgent need to strengthen mental health systems^54^.

Reproductive and maternal healthcare was generally more resilient compared to other services. Only two health systems— Chile and Mexico’s public sectors—had large declines in contraceptive provision (52% and 87%, respectively). Although some public sector users may have switched to the private sector or to pharmacies for contraceptives, the unmet need for contraception appears to have increased across Latin America and the Caribbean during the COVID-19 pandemic^55^. Frequent contraceptive shortages were also reported. By contrast, family planning visits declined by only 4% and 14% in Nepal and Haiti, respectively, despite large disruptions in other services. This finding is consistent with other studies from low- and lower-middle-income countries (including from household surveys) that found relatively small changes in use of family planning services during the pandemic^11,13,15,16^. Economic uncertainties during the pandemic may also have led to an increased demand for contraception^15^.

The number of facility-based deliveries declined substantially in Haiti, Nepal, Mexico and South Africa but were relatively stable in the other six countries reporting. Other studies also found mixed results for the effect of the pandemic on facility-based deliveries^11,13,14,16,24,56^. Reasons for this likely vary by country. At the Mexican IMSS, many hospitals were converted into COVID-19 treatment centers, and many pregnant women were redirected to the private sector for childbirth (sometimes at their own cost)^44^. In South Africa, the dataset contained information from all public and private hospitals (in the KwaZulu-Natal province only). Thus, the 11% decline in facility-based deliveries likely reflects an increase in home births. Ethiopia, one of the countries with the lowest rate of facility deliveries, had a 3% decline in facility deliveries (not statistically significant). However, this estimate likely hides sub-national disruptions. One study using household survey data found a decline in hospital births in urban areas only^57^. Similarly in Haiti and Nepal, more women might have opted to give birth at home or with traditional attendants^58^. This could be associated with an increase in maternal and perinatal mortality and morbidity^9,59^. Poorer antenatal care follow-up could also lead to a higher number of pre-term births and stillbirths^60,61^.

Visits for children younger than 5 years of age with diarrhea and pneumonia declined in all countries reporting, which was also reported by others^13,16^. Part of these declines may be explained by a reduced incidence of diarrhea and pneumonia from social distancing, school and daycare closures, mask wearing and improved handwashing practices^62^. Some caregivers may have also opted to seek treatment from pharmacies, shops or the informal sector for their children’s illness rather than visit health facilities, which would not be reflected in our data.

Health system design and organization before the pandemic may be associated with health service resilience. For example, in Chile, maternal health services are provided exclusively by midwives who were not redeployed to COVID-19 care and were able to maintain regular service provision^63^. In South Korea, the number of hospital beds per capita is about three times higher than the Organization of Economic Cooperation and Development average^64^. Thus, the country may have been able to reallocate a large share of this capacity to COVID-19 care without substantial negative effects on other services. South Korea also benefited from prior investments and a stronger public health response system, given its experience handling the SARS outbreak of 2003, the novel influenza outbreak of 2009 and the MERS-CoV epidemic of 2015 (ref. ^27^). The private sector in Mexico is large and expanding, and private facilities were able to provide maternity care for a high percentage of public sector users while public hospitals were repurposed to COVID-19 care^44^.

Our analysis has several strengths. We estimated the effect of the COVID-19 pandemic on 31 health services using administrative and RHIS data that represented the complete, or nearly complete, census of all health facilities in the country (or province in the case of Kwa-Zulu Natal in South Africa). Unlike costly population health surveys, administrative and RHIS data can provide near real-time data on the performance of health systems. Our study also included countries from all income groups, which provides a more comprehensive picture of the effects of the pandemic. Nonetheless, our study has limitations. First, although we included a range of countries, our results cannot be generalized to their regions or to other parts of the world. Second, the number and type of indicators available in each country varied, including slight variations in definitions (Supplementary Table 1). Thus, cross-country comparisons should be made with care. Third, the exclusion of private providers in some countries in this analysis limits our ability to quantify the extent to which patients switched from the public sector to the private sector for healthcare during the pandemic. Similarly, the routine data systems generally did not include telemedicine consultations that were made during the pandemic. Fourth, disruptions were assessed only at the national level, and our estimates could hide disruptions that occurred in specific sub-national regions, cities, types of health facilities or population groups within a country. Fifth, it is possible that the pandemic affected the quality of reporting in administrative sources and RHIS. However, we used thorough data-cleaning procedures and only used data from facilities that continuously reported throughout the study period in the six countries with disaggregated data (Chile, Ethiopia, Haiti, Laos, Nepal and South Africa). Sixth, our main analysis covers only the first 9 months of the pandemic. However, data for the first 6 months of 2021 in a subset of countries reveal that service disruptions continued in many countries in 2021.

Our findings have implications for current health system planning and for the management of future pandemics. Despite the many efforts deployed to maintain the continuity of health services, we found considerable declines in healthcare use. Part of these declines may be linked to decreased healthcare needs during the pandemic from reductions in non-COVID-19 infectious illnesses and fewer injuries. Nonetheless, a larger share of these declines likely reflects a failure of health system resilience. Health systems must urgently resume essential care and plan to compensate for missed needed services. This includes catching up on missed preventive care (such as health screenings and immunizations) and identifying and addressing any adverse health consequences of missed services, such as trauma care, surgery, C-sections and chronic disease management. These can include physical sequelae (for example, obstetric fistulae), chronic disease complications and health-related suffering. Higher rates of uncontrolled hypertension and diabetes, for example, could lead to an increase in cardiac events and in complications of diabetes, such as blindness, kidney failure and lower limb amputations^2^. In Chile and Mexico, the decline in family planning could result in higher rates of unplanned pregnancies. Finally, the pandemic’s negative effects on mental health, combined with declines in in-person care, will lead to greater unmet needs for mental healthcare and a potential increase in suicides^53^. Increased investments in health systems are needed to address these consequences and the surge in pent-up demand as well as to prepare the health systems for more agile function in the future. Given limited resources in some countries that will be further strained by the global economic downturn due to the COVID-19 pandemic, priority should be given to health interventions that will have the greatest benefits on health^65^.

Further research is needed to understand the full indirect health effects of the pandemic and the factors responsible for service disruptions. For example, our analysis did not assess changes in the quality of healthcare, which was likely affected during the pandemic (including from poorer processes of care and shortages of medicines or supplies), resulting in poorer health outcomes even among those who received care^7^. Similarly, future research should monitor trends in mortality from non-COVID-19 conditions and assess whether certain population subgroups (such as ethnic minorities, teenagers or the poorest) were differentially impacted by health service disruptions. Finally, it is important to disentangle the factors responsible for disruptions in health services.

In 2021, nearly all countries included in this analysis experienced larger waves of SARS-CoV-2 infections and often re-implemented periodic lockdowns, including to prevent further spread of the Delta variant^4^. Given the widespread disruptions in health services demonstrated in this paper, many of which were unrelated to COVID-19 severity, our results call for rethinking pandemic preparedness and health system response. The unintended consequences of COVID-19 responses may have outweighed the loss of life from COVID-19 itself, particularly in LMICs^66^. Health system resilience must become a central component of national health plans. Given the likelihood of future pandemics and other major shocks, there is an urgent need to design more resilient health systems capable of addressing a crisis while maintaining essential functions.

## Methods

### Data sources

This research was undertaken as part of the QuEST network, a new initiative focused on increasing the impact and scale of research on health systems through multi-country partnerships^19^. As per the QuEST approach, we aimed for representation of countries from different regions and with different income levels, health system types and severity of COVID-19. The initial outreach was to researchers and policymakers with past collaborations on health system research (including the Lancet Global Health Commission on High Quality Health Systems)^2^. Authors initially invited potential collaborators from 18 countries; collaborators in ten countries were able to assemble the required data (Supplementary Table 6).

The institutional review board of the Harvard T. H. Chan School of Public Health determined this study as exempt from full review.

We used administrative and RHIS data reported monthly by health facilities. In six countries (Ethiopia, Ghana, Haiti, Lao People’s Democratic Republic, Nepal and South Africa), RHIS data were extracted from the DHIS2 platform. The DHIS2, formerly known as the district health information system-2, is the largest web-based RHIS platform in the world and is currently used in over 70 LMICs^77^. In Chile, Mexico, South Korea and Thailand, the data were obtained from various national administrative health information systems, described in Table 1. Local investigators led local data extractions, which began in April 2020 and ended in August 2021.

In four countries (Ghana, Haiti, Nepal, South Korea), these data represented all health facilities in the country. In Ethiopia, the data included all health facilities in the country except for those in the Tigray region which was excluded due to the conflict in the region which began in the last months of 2020. In Lao PDR, Thailand and Mexico, the data included only public sectors which covered services provided to approximately 50%-70% of the population. In Chile, certain indicators were available from both public and private hospitals (deliveries, C-sections, inpatient admissions, hospital discharges for child pneumonia, and road traffic accidents) while other indicators included only the public sector. In South Africa, the data were from all facilities in the KwaZulu-Natal province.

### Study design and timeline

We used a prospective single interrupted time series design with data before and after the declaration of the pandemic^21^. This approach allows each country to act as its own control (the pre-pandemic period being the control). We collected routine monthly data on health service delivery for the period of 1 January 2019 to 31 December 2020 (24 months). In Ethiopia, the data were collected from Tahsas 2011 to Tahsas 2013 (equivalent to 30 December 2018 to 29 December 2020). In Nepal, the data were collected from Magh 2075 to Poush 2077 (equivalent to 15 January 2019 to 13 January 2021).

The World Health Organization declared COVID-19 a pandemic on 11 March 2020 (ref. ^21^). In addition to the declaration of the pandemic, major lockdowns and containment policies (for example, restricted movements, public transport closures and curfews) were put in place in most countries by the end of March 2020. For our analysis, the pre-pandemic (baseline) period includes 15 months, from January 2019 to March 2020, and the pandemic period includes 9 months, from April to December 2020. In Nepal, the pre-pandemic period includes 14 months, and the COVID-19 period includes 10 months.

### Outcome measures

We aimed to include health services for a wide range of conditions facing different age groups. The list of conditions addressed by the Sustainable Development Goals formed the basis for selection (Supplementary Table 7). Within data availability constraints, we assessed use of a large range of health services, including reproductive, maternal and child health services (including routine child vaccinations); HIV, TB and malaria services; chronic disease care; and road traffic injuries. We also included five summative measures of health system contacts: total outpatient visits, emergency room visits, inpatient admissions, surgeries and trauma admissions. We only included indicators that were reported monthly. For example, data on TB care in Ethiopia were excluded because they were available only quarterly. Certain indicators were also excluded due to poor data quality (see ‘Data cleaning’ section below). In total, 31 different health services were included in the final analyses, including between seven and 22 per country (with a median of 18 indicators per country). Detailed definitions are shown in Supplementary Table 1. We analyzed the absolute number of visits or services provided rather than service coverage indicators (that is, proportion of target population who received a specific service) as the latter can be unreliable because they depend on estimated target population sizes (for example, facility catchment population) as denominators^78,79^.

### Level of aggregation

The raw RHIS data were obtained at different levels of aggregation. In Ghana, the data were obtained at the regional level, in Mexico at the delegation level (with one delegation per state except in Mexico City, the State of Mexico and Veracruz with two delegations per state) and in Thailand and South Korea at the provincial level.

In Ethiopia, the dataset combined facility-level data and woreda-level data (a woreda is the smallest administrative subdivision in Ethiopia): facility-level information was available for tertiary and secondary hospitals and private facilities. Data from other public facilities (primary hospitals, health centers and health posts) were aggregated and reported by woreda health offices. In Nepal, RHIS data were obtained at the palika level (the smallest administrative subdivision in Nepal). In Chile, all indicators were at the health facility level except vaccinations (available at the commune level) and breast cancer screening and child pneumonia, which were available at the region level only. In Haiti, Lao People’s Democratic Republic and South Africa, the data were available at health facility levels, the lowest possible level for these data.

### Data cleaning

A potential source of bias in any ITS analysis relates to the potential effect of the intervention (in our case, the pandemic) on data collection and reporting^20^. As health systems prepared for COVID-19 or, in some countries, were faced by large waves of COVID-19 cases, efforts to report to the RHIS may have been affected. In addition, concerns about RHIS data quality remains in several lower-income countries^78,80^. To address these concerns, we conducted thorough data cleaning in the six health systems with facility-level or municipality-level information (Chile, Ethiopia, Haiti, Laos, Nepal and South Africa). In the other four countries (Ghana, Mexico, South Korea and Thailand), data cleaning was performed by data custodians^81,82^.

We conducted a two-step cleaning procedure. First, the primary concern with using RHIS during the pandemic is that reporting completeness may decrease if health facilities delay or omit monthly RHIS reporting. Poorer reporting during the pandemic would bias effect estimates upward if non-reporting is mistaken for a reduction in service use. Our cleaning procedure addressed this concern by including only health facilities that reported each indicator for at least 15 months out of 24. This led to stable numbers of facilities reporting each month. We tested a range of more- and less-stringent thresholds for inclusion across the six countries (Supplementary Table 8). Results were largely similar using the 12-, 15- or 18-months thresholds. The complete cases analysis requiring all 24 months excluded too much data and led to biased results. The 15 months out of 24 thresholds balanced the needs for complete reporting and representativeness. Second, we identified outliers, defined as service counts greater than 3.5 standard deviations from the facility mean over 24 months, and set the values to missing^83^. Low outliers (values lower than 3.5 standard deviations from the mean), were not removed, as service use may have legitimately decreased to low levels during the pandemic. Finally, we compared datasets before and after these two steps to ensure that data cleaning did not bias the datasets. We compared the sum of each indicator in the raw versus cleaned dataset to ensure that cleaning procedures did not exclude too much data, rendering the dataset non-representative (Supplementary Table 9).

### Statistical analysis

We used an interrupted time series analysis of each country’s data to estimate the impact of the pandemic on health service use. We propose an impact model where the pandemic would have an immediate short-term effect (over 6 months) on the level and trend in health services, followed by a potential resumption in the last quarter of 2020: a return to the pre-pandemic level and trend.^72^ Number of healthcare visits was modeled separately for each service using fixed effects ordinary least square segmented regression analysis. All analyses were conducted at the level of the sub-national units shown in Table 1 (that is, region, provinces, delegations or districts). Standard errors were clustered at the same level to account for serial correlation^84^. Because there were relatively few sub-national units in some countries, we further presented *P* values and 95% confidence intervals based on critical values from a *t*-distribution with G-2 degrees of freedom, where G is the number of sub-national units^85,86^. The interrupted time series regression model used the equation:

$$Y_{it} = \beta _0 + \beta _1T + \beta _2X_t + \beta _3Z + \beta _4W + \beta _5S + \beta _6R + {\it{\epsilon }}_{it}$$

Where *Y*_*it*_ represents the number of patient visits in sub-national unit *i* during month *t*; *T* represents the number of months since January 2019; *X*_*t*_ is an indicator variable that equates 1 for the 6 months after the declaration of the pandemic; *Z* represents the number of months since the pandemic was declared from 1 to 6 (and equates to 0 for the pre-pandemic period); *W* is an indicator variable that equates 1 for the potential resumption period (that is, the last quarter of 2020); *S* is a vector of dummy variables for each of the four seasons (spring, summer, fall and winter); *R* is a vector of dummy variables for each region (or other country-specific subnational unit as described in the last column of Table 1); and ${\it{\epsilon }}_{it}$ is the random error term. Here, *β*_0_ represents the baseline level of the health service; *β*_1_ is the change in health service volume each month pre-pandemic; *β*_2_ represents the change in the level of the health service after the pandemic was declared; *β*_3_ is the difference in the slope post-intervention compared to the pre-intervention period; *β*_4_ represents the remaining level change during the last quarter of 2020; *β*_5_ is the change in health service volume according to the season; and *β*_6_ is the change in service volume by sub-national unit (region, province or district). *β*_2_ and *β*_4_, our primary parameters of interest, are expressed in the main text in relative terms as the percentage change from the average in the pre-COVID-19 period. Absolute values, along with other coefficients from the regression models, are reported for each country in Supplementary Tables 10–19.

Finally, to estimate the burden of missed healthcare across the ten countries, we provide estimates of the cumulative number of visits missed for three groups of services: maternal and neonatal care, chronic disease care and childhood vaccination. For each service, we predicted what service delivery would have been in each post-pandemic month had the pre-pandemic trend continued and subtracted that from the observed value after adjusting for seasonality. We summed missed visits across all months from April to December 2020. To make the estimates comparable across populations, we divided the sum of missed chronic disease care by the total population of the country (or province for KwaZulu-Natal) and the sum of missed maternal, newborn and child care visits by the number of annual births, estimated by multiplying the population by the crude birth rate (Supplementary Table 20). All analyses were performed using STATA version 16.

### Reporting Summary

Further information on research design is available in the Nature Research Reporting Summary linked to this article.

## Online content

Any methods, additional references, Nature Research reporting summaries, source data, extended data, supplementary information, acknowledgements, peer review information; details of author contributions and competing interests; and statements of data and code availability are available at 10.1038/s41591-022-01750-1.

## Supplementary information

Supplementary Information Supplementary Tables 1–20

Reporting Summary

## Data Availability

The data used in this study were collected from multiple sources. In Chile, data are publicly available from https://deis.minsal.cl/. The data from the Mexican Institute for Social Security were deposited in a repository.^87^ In Thailand, data are publicly available from the Ministry of Public Health: http://hdcservice.moph.go.th/. In all other countries, the data are restricted, and permissions to access the data must be obtained from the respective Ministries of Health.

## Code availability

All code supporting this analysis can be accessed from https://github.com/catherine-arsenault/HS-performance-during-covid-do-files/tree/master/Multi-country/Paper%201%20Service%20utilization.
