# Supplementary material for: COVID-19 and resilience of healthcare systems in ten countries
Source: Nat Med. 2022 Mar 14;28(6):1314–24. doi: 10.1038/s41591-022-01750-1 (PMC9205770; doi:10.1038/s41591-022-01750-1)
Supplement: Supplementary file 1 — Supplementary Tables 1–20 [file 41591_2022_1750_MOESM1_ESM.pdf]

nature portfolio

https://doi.org/10.1038/s41591-022-01750-1

Supplementary information

COVID-19 and resilience of healthcare systems in ten countries

In the format provided by the authors and unedited

COVID-19 and resilience of healthcare systems in 10 countries
Arsenault C, Gage A, Kim MK, et al.

Supplementary tables

Supplementary table 1. Indicator definition by country 2
Supplementary table 2. Estimated number of health care visits and vaccinations missed from April to December 2020 in 10 countries 5
Supplementary table 3. COVID incidence, the stringency index and health service disruptions, by country 6
Supplementary table 5. Country-specific factors promoting and disrupting health services during the pandemic 8
Supplementary table 6. Characteristics of countries included 12
Supplementary table 7. Health conditions addressed by the Sustainable Development Goals (SDGs) and covered in the analysis 13
Supplementary table 8. Sensitivity analysis using different reporting thresholds for inclusion during data cleaning 14
Supplementary table 9. Number of health care visits included in the datasets from January 2019 to December 2020 in six countries, before and after data cleaning 17
Supplementary tables 10-19. Estimates from segmented regression analyses for the impact of the pandemic on health service levels in 2020 19
- Chile 19
- Ethiopia 20
- Ghana 21
- Haiti 22
- Lao, PDR 23
- Mexico, IMSS 24
- Nepal 25
- South Africa 26
- South Korea 27
- Thailand 28

Supplementary table 20. Population and birth estimates used for calculations of missed health care visits per 1,000 29

Supplementary table 1. Indicator definition by country

		Chile	Ethiopia	Ghana	Haiti	Lao PDR	Mexico	Nepal	South Africa	South Korea	Thailand
**Summative measures**	**Outpatient visits**		Number of outpatient visits	Total OPD attendance	Visites des Clientes PF | Repartition des vistes +<br>Visites des Enfants 1 - 4 ans | Repartition des visites +<br>Visites des Enfants 10 - 14 ans | Repartition des visites +<br>Visites des Enfants 5 - 9 ans | Repartition des visites +<br>Visites des Enfants < 1 an | Repartition des visites +<br>Visites des Femmes Enceintes | Repartition des visites +<br>Visites des Jeunes adultes 15 - 19 ans | Repartition des visites +<br>Visites des Personnes a mobilite reduite (moteur) +<br>Visites des Personnes a mobilite reduite (sensoriel) +<br>Visites des jeunes adultes 20 - 24 ans | Repartition des visites +<br>Vistes des Autres Adultes | Repartition des visites	OPD: Outpatient visits	Number of visits to family medicine clinics +<br>Number of outpatient specialty consultations	Disaggregation by Sex & Caste/Ethnicity - Outpatient Cases	Total clients attending general or specialist Outpatient clinics	Number of outpatient visits	OPD visit
	**Emergency room visits**	Number of emergency visits	Total number of emergency unit attendances				Number of emergency room visits	Clients Received Emergency Services - Age Group 0-9 Years +<br>Clients Received Emergency Services - Age Group 10-19 Years +<br>Clients Received Emergency Services - Age Group 20-59 Years +<br>Clients Received Emergency Services - Age Group >-60 Years		Number of emergency room visits	
	**Inpatient admissions**	Egresos totales	Number of inpatient admissions	Total Admissions		IPD: Inpatient visits	Number of inpatient admissions totals	Inpatient Morbidity Cases	Admissions - Total	Number of inpatient admissions totals	IPD visit
	**Surgeries**	Number of surgical operations									
	**Trauma admissions**								Admissions - trauma		
**Reproductive and maternal**	**Family planning**	Number of contraceptive users	Total new and repeat acceptors disaggregated by method	Number of new and current users of contraceptives	Visites des Clientes PF | Repartition des vistes	Combined pill New user<br>Combined pill Continue user<br>Emergency pill New user<br>Emergency pill Continue user<br>Single pill new users<br>Single pill Continue users<br>Depose (Injectable) New users<br>Depose (Injectable) Continue users<br>Condoms New users<br>Condoms Continue users	Number of new users of contraceptives +<br>Number of current users of contraceptives	Family Planning Program - Temporary FP Method - Depo-Current User +<br>Family Planning Program - Temporary FP Method - Depo-New Users < 20 Years +<br>Family Planning Program - Temporary FP Method - Depo-New Users > 20 Years +<br>Family Planning Program - Temporary FP Method - Pills-Current User +<br>Family Planning Program - Temporary FP Method - Pills- < 20 Years +<br>Family Planning Program - Temporary FP Method - Pills- > 20 Years +<br>Safe Motherhood Program-Safe Abortion Service-Post Abortion FP Methods Short Term-Medical +<br>Safe Motherhood Program-Safe Abortion Service-Post Abortion FP Methods Short term-Surgical +			
	**Antenatal care**	Number of antenatal care attentions made at a health facility	Number of pregnant women that received ANC at least once by maternal age	ANC attendance	Visites des Femmes Enceintes | Repartition des visites	ANC 1st visit by Health facility/Outreach	Number of first antenatal care visits +<br>Number of subsequent antenatal care visits<br>(or number of total antenatal care visits)	Safe Motherhood Program-Antenatal Checkup-First ANC visits (any time) < 20 years +<br>Safe Motherhood Program-Antenatal Checkup-First ANC visits (any time) > 20 years	Number of first antenatal care visits total	Number of antenatal care visits	
	**Deliveries**	Partos totales	Total number of births attended by skilled health personnel	Number of births attended by skilled health personnel at a health facility	Accouchements Institutionnels	Delivery at HF	Number of facility deliveries	Safe Motherhood Program-Delivery Service-Skilled Birth Attendants (SBA)Facility	Delivery in facility sum	Number of facility deliveries	Total delivery cases
	**Caesarean sections**	Partos cesareas	Number of women having given birth by caesarean section	Caesarean section deliveries		Caesarean delivery	Number of caesarean sections	Safe Motherhood Program- Type of Delivery - C/S Breech +<br>Safe Motherhood Program- Type of Delivery - C/S Cephalic +<br>Safe Motherhood Program- Type of Delivery - C/S Shoulder	Delivery by caesarean section	Number of caesarean sections	

		Chile	Ethiopia	Ghana	Haiti	Lao PDR	Mexico	Nepal	South Africa	South Korea	Thailand
**Child health**	**Postnatal care**	Number of postnatal visits up to 10 days of life	Number of postnatal visits within 7 days of delivery	1st PNC on day 1 or 2 +<br>1st PNC on day 3-7	Consultations postnatales	PNC within 2 days +<br>PNC 3-42 days		Safe Motherhood Program- 3 PNC visits as per protocol	Infant postnatal visit within 6 days after delivery		
	**Diarrhea**		Number of children treated for diarrhea with ORS only	Diarrhea Diseases (Under 5 years)			Number of consultations for sick child care – diarrhea	CBIMCI-(2-59Months)- Classification-Diarrhea-Dysentery +<br>CBIMCI-(2-59Months)- Classification-Diarrhea-No Dehydration +<br>CBIMCI-(2-59Months)- Classification-Diarrhea-Prolonged Diarrhea +<br>CBIMCI-(2-59Months)- Classification-Diarrhea-Severe Dehydration +<br>CBIMCI-(2-59Months)- Classification-Diarrhea-Some Dehydration	Child under 5 years with diarrhoea as main final diagnosis at separation	Number of consultations for sick child care – diarrhea	
	**Malnutrition**		Total number of children <5yrs screened for acute malnutrition				Number of consultations for sick child care – malnutrition				
	**Pneumonia**	Number of hospital discharges for children under 5 with pneumonia	Number of children under 5 treated for pneumonia	Pneumonia Cases (Under 5 years)			Number of consultations for sick child care – pneumonia	CBIMCI-(2-59Months)- Classification-ARI-Pneumonia +<br>CBIMCI-(2-59Months)-ORC Classification-ARI-Severe Pneumonia/Very Severe Disease	Child under 5 years classified as pneumonia according to the IMCI definition	Number of consultations for sick child care – pneumonia	
**Child vaccinations**	**BCG**	BCG	Number of children under one year of age who have received BCG vaccine	Number of children immunized by age 1 -BCG		BCG	Number of children vaccinated with a unique dose of BCG	Immunization program - Vaccine Type- Children Immunized - BCG Doses	BCG (tuberculosis) vaccine given to a child under one year of age at birth.		
	**Pentavalent**	Hexavalent vaccination	Number of children under one year who have received third dose of pentavalent vaccine	Number of children immunized by age 1 - Penta 3		Penta 3	Number of children vaccinated with 3rd dose pentavalent +<br>Number of children vaccinated with 3rd dose of pentavalent acellular	Immunization program - Vaccine Type- Children Immunized - DPT-HepB-Hib 3rd	DTaP-IPV Hib-HBV (Hexavalent) 3rd dose given to a child under one year		
	**Pneumococcal**	Pneumococcal vaccination	Number of children under one year of age who have received third dose of pneumococcal vaccine	PCV 3 - Children Vaccinated		PCV 3	Number of children who received the third dose of pneumococcal conjugate vaccine	Immunization program - Vaccine Type - Doses Children Immunized - PCV-3rd	Pneumococcal (PCV) vaccine 3rd dose given to a child under one year		
	**Rotavirus**		Number of children under one year of age who have received 2nd dose of Rotavirus vaccine	Rotavirus 1 - Children Vaccinated +<br>Rotavirus 2 - Children Vaccinated			Number of children who received the second dose of the rotavirus vaccine		Rota Virus (RV) vaccine 2nd dose given to a child under one year		
	**Measles**	Tres Virica vaccination (measles, mumps, and rubella)	Number of children under one year who received first dose of measles vaccine	Measles Rubella 1 - Children Vaccinated<br>+ Measles Rubella 2 - Children Vaccinated			Number of children vaccinated with a second dose of the Triple Viral MMR (measles, mumps, and rubella) vaccine	Immunization program - Children Immunized - Measles/Rubella - 9-11 Months + Immunization program - Children Immunized - Measles/Rubella - 12-23 Months	Measles vaccine 2nd dose given to a child at 12 months after birth		
	**Fully vaccinated by 1**		Number of children received all vaccine doses before 1st birthday	Fully immunized children 0-11m+<br>Fully immunized children 12-23 months	Complètement Vaccinés Communautaires +<br>Completement Vaccinés Institutionnelles				Immunized fully under one year new		
**HIV, TB and malaria**	**People on ART**		Number of adults and children who are currently on ART				Number of adult and children receiving ART		Total clients remaining on ART (TROA) end of month - sum	Number of adult and children receiving ART	
	**HIV tests**							Virology-HIV tests conducted			
	**TB screening**								Clients 5 years and older who were screened in health facilities for TB symptoms using the standard TB screening tool as per National TB Guideline		
	**TB detection**			Number of TB cases detected				Disaggregation by Sex & Caste/Ethnicity- New TB Cases	Clients 5 years and older who were confirmed with Drug-Susceptible TB (DS-TB) diagnosis		
	**TB treatment**								Clients 5 years and older who were started on TB treatment regimen		
	**Malaria visits**			Number of OPD Malaria cases							Malaria cases
**Chronic disease and road traffic accidents (RTA)**	**Diabetes visits**	New patients enrolled in the diabetes program		Diabetes Mellitus	Anciens Cas Diabete+<br>Nouveaux Cas Diabete	OPD Diabetes	Number of diabetic patients visited primary care clinics (20+ years)	Outpatient Morbidity-Nutritional & Metabolic Disorder-Diabetes Mellitus (DM) Cases	Diabetes treatment visit (Every visit for routine care by clients 40 years and older on treatment for diabetes)	Number of diabetic patients visited facility during the reporting period	Diabetes cases
	**Hypertension visits**	New patients enrolled in the cardiovascular program		Hypertension	Anciens Cas HTA +<br>Nouveaux Cas HTA	OPD Hypertension	Number of hypertensive patients visited primary care clinics (20+ years)	OPD-Morbidity-Cardiovascular & Respiratory Related Problems-Hypertension		Number of hypertensive patients visited	New hypertension cases

		Chile	Ethiopia	Ghana	Haiti	Lao PDR	Mexico	Nepal	South Africa	South Korea	Thailand
										facility during the reporting period	
	**Cervical cancer screening**						Number of women aged 25 - 64 screened with VIA for cervical cancer for the first time		Cervical cancer screening (pap smear, visual inspection with Acetic acid (VIA) and liquid base cytology) for women 30 years and older		
	**Breast cancer screening**	Mammographies					Número de mujeres entre 50 y 69 años con mastografía de tamizaje de Cáncer de Mama, de primera vez				
	**Mental health care**	Number of mental consultations					Number of consultations for attempted suicide			Number of mental health consultations	
	**Road traffic accidents**	Number of traffic injuries	Number of road traffic injury cases disaggregated by accident type	Transport injuries (Road Traffic Accidents)		OPD Road traffic injury + IPD Road traffic injury			Emergency case- Motor Vehicle Accident - Occupant + Emergency case - Motor Vehicle Accident - Pedestrian		Number of traffic accident cases

Supplementary table 2. Estimated number of health care visits and vaccinations missed from April to December 2020 in 10 countries

		Chile	Ethiopia	Ghana	Haiti	Lao PDR	Mexico	Nepal	South Africa	South Korea	Thailand
**Antenatal care visits**	Estimate	3,472*	41,930*	10,272*	17,500*	-6,318*	664,125*	56,210*	-12,969*	-47,566*	
	(95% CI)	(4,674, 2,270)	(53,483, 30377)	(18,641, 1,903)	(25,853, 9,147)	(-5,925, -6,711)	(668,572, 659,678)	(57,598, 54,822)	(-11,600, -14,338)	(-34,728, -60,404)	
**Postnatal care visits**	Estimate	-224	14,980*	-44,224*	19,130*	-3,546*		7,084*	13,519*		
	(95% CI)	(619, -1,067)	(28,390, 1,570)	(-42,022, -46,426)	(19,994, 18,266	(-2,862, -4,230)		(7,621, 6547)	(14,169, 12,869)		
**Institutional deliveries**	Estimate	-5824*	-9,610*	11,520*	19,930*	-7,236*	32,200*	55,825*	10,956*	-4,437*	-2,464*
	(95% CI)	(-4,599, - 7,049)	(-3,480, - 15,740)	(18,381, 4,659)	(20,775, 19,085)	(-6,946, -7,526)	(32,921, 31,479)	(56,866, 54,784)	(11,558, 10,354)	(-3,463, -5,411)	(-1,571, -3,357)
**BCG vaccinations**	Estimate	1,952*	-128,450*	-65,536*		-4,338*	116,690*	21,868*	27,423*		
	(95% CI)	(2,688, 1,216)	(-119,314, - 137,586)	(-61,442, -69,630)		(-3,800, -4,876)	(117,992, 115,388)	(23,417, 20,319)	(28,497, 26,349)		
**Pentavalent vaccinations**	Estimate	9,120*	12,980*	-50,784*		-1,818*	96,110*	8,701*	4,741*		
	(95% CI)	(9,972, 8,268)	(24,430, 1,530)	(-46,472, -55,096)		(-1,344, -2,292)	(99,187, 93,033)	(9,733, 7,669)	(5,304, 4,178)		
**Pneumococcal conjugate vaccinations**	Estimate	19,440*	11,790*	-17,504*		-1,548*	7,735*	2,695*	16,533*		
	(95% CI)	(20,074, 18,806)	(22,896, 684)	(-13,737, -21,271)		(-1,077, -2,019)	(8,074, 7,396)	(3,290, 2,100)	(16,915, 16,151)		
**Measles vaccinations**	Estimate	19,152*	-16,180*	-10,400*			-62,335*	6,237*	12,936*		
	(95% CI)	(19,867, 18,437)	(-6,705, - 25,655)	(-1,433, -19,367)			(-60,822, -63,848)	(7,346, 5,128)	(13,657, 12,215)		
**Diabetes consultations**	Estimate	16,688*		-15,200*	14,210*	-18	4,154,430*	144,529*	267,916*	-183,872*	11,473*
	(95% CI)	(17,786, 15,590)		(-13,514, -16,886)	(15,282, 13,138)	(1,001, -1,037)	(4,193,690, 4,115,170)	(146,602, 142,456)	(273,613, 262,219)	(-139,373, -228,371)	(11,890, 11,056)
**Hypertension consultations**	Estimate	38,368*		816	60,010*	7,020*	1,975,540*	258,027*		-188,394*	32,263*
	(95% CI)	(39,918, 36,818)		(6,856, -5,224)	(63,758, 56,262)	(7,658, 6,382)	(2,026,341, 1,924,739)	(261,520, 254,534)		(-121,903, -254,885)	(33,286, 31,240)

Asterisks * indicates statistical significance ($p<0.05$). Negative numbers indicate that more visits took place than expected according to pre-Covid trend. Positive numbers are the number of missed visits.

Supplementary table 3. COVID incidence, the stringency index and health service disruptions, by country

Country	Cumulative COVID cases per million population by September 30, 2020 [b]		Stringency index (average from April 1 to September 30, 2020) [c]		Remaining level change in outpatient visits by October 2020		Remaining level change in deliveries by October 2020		Remaining level change in inpatient admissions by October 2020		Remaining level change in pentavalent vaccinations by October 2020		Remaining level change in diabetes visits by October 2020	
	Number	Rank	Number	Rank	Estimate	Rank	Estimate	Rank	Estimate	Rank	Estimate	Rank	Estimate	Rank
Chile	24,099	1	79.85	2	-	-	7.38	9	-2.35	8	-1.49	2	5.18	7
South Africa [a]	11,231	2	78.38	4	-20.75	4	-11.41	4	-12.24	4	-1.40	3	-33.01	2
Mexico	5,706	3	75.22	5	-33.45	1	-26.33	1	-27.14	2	-12.86	1	-32.74	3
Nepal	2,622	4	86.30	1	-22.40	2	-22.07	2	-60.60	1	11.04	6	-59.20	1
Ghana	1,469	5	57.14	8	-6.64	7	-1.35	6	-2.72	7	8.47	5	20.00	9
Haiti	757	6	61.25	6	-3.93	9	-15.46	3	-	-	-	-	-26.59	4
Ethiopia	639	7	79.72	3	-7.00	6	2.32	8	-15.48	3	-0.74	4	-	-
South Korea	466	8	53.56	9	-5.32	8	0.95	7	-3.29	6	-	-	0.62	6
Thailand	51	9	60.75	7	-21.99	3	-6.20	5	-10.22	5	-	-	12.65	8
Lao PDR	3	10	50.19	10	-12.67	5	13.07	10	23.74	9	12.79	7	-4.86	5

Countries are sorted by COVID incidence. Ranks are by highest COVID incidence, highest number of COVID tests, highest stringency of COVID response measures and by largest disruptions in health services. Pairwise correlation coefficients between cumulative COVID incidence and disruptions in health services are -0.53 for outpatient visits, 0.12 for deliveries, 0.03 for inpatient admissions, -0.40 for pentavalent vaccinations, and 0.02 for diabetes visits. Pairwise correlation coefficients between the average stringency index and disruptions in health services are -0.50 for outpatient visits, -0.45 for deliveries, -0.73 for inpatient admissions, -0.44 for pentavalent vaccinations, and -0.65 for diabetes visits.

[a] In South Africa, the analysis only included the KwaZulu-Natal Province and does not represent national-level disruptions. COVID cases and stringency are at the national level.
[b] COVID cases are from the COVID-19 Data Repository by the Center for Systems Science and Engineering (CSSE) at Johns Hopkins University
[c] The stringency index was developed by the Oxford COVID-19 Government Response Tracker. The stringency index is a composite measure based on nine response indicators including school closures, workplace closures, cancellation of public events, restrictions on public gatherings, closures on public transport, stay-at-home requirements, public information campaigns, restrictions on internal movements and international travel controls, rescaled to a value from 0 to 100 (100 = strictest).

Supplementary table 4. Immediate effect of the pandemic on five health services at the national level and in the largest metropolitan region in each country

		Percent change in outpatient visits from April to September 2020		Percent change in deliveries from April to September 2020		Percent change in inpatient admissions from April to September 2020		Percent change in pentavalent vaccinations from April to September 2020		Percent change in diabetes visits from April to September 2020	
**Country/ Province**	**Metropolitan region**	National level	Metropolitan region only	National level	Metropolitan region only	National level	Metropolitan region only	National level	Metropolitan region only	National level	Metropolitan region only
Chile	Región Metropolitana de Santiago	-	-	1.13	-35.15	-46.69	-49.46	-19.77	-28.96	-80.24	-85.74
Ethiopia	Addis Ababa region	-23.62	-50.76	-2.69	2.56	-31.76	-37.35	-4.01	-4.21	-	-
Ghana	Greater Accra region	-19.84	-29.50	6.92	3.32	-15.15	-17.58	5.16	7.98	-6.54	-30.55
Haiti	Département de l'Ouest	-32.37	-36.81	-31.09	-35.64	-	-	-	-	-61.99	-96.23
Lao PDR	Vientiane Capital province	-32.33	-33.73	-4.69	5.89	-36.78	-35.08	-24.17	-21.37	1.10	-10.56
Mexico	DF Norte & DF Sur	-40.50	-46.09	-2.17	-1.69	-59.85	-61.86	-43.22	-57.27	-26.24	-33.00
Nepal	Kathmandu District	-17.82	-25.56	-11.63	-16.94	-39.02	-45.99	-49.28	-63.01	-57.59	-71.32
South Africa[a]	eThekwini Metropolitan District	-31.06	-37.84	-20.62	-26.32	-26.99	-34.42	-14.91	-34.22	-23.80	-33.24
South Korea	Seoul district	-9.38	-8.81	-0.50	4.05	-3.82	-1.27	-	-	-2.90	-1.54
Thailand	Bangkok province	-32.34	15.00	5.70	11.67	-31.13	-46.17	-	-	-	-

Estimates are expressed as the % change after the declaration of the pandemic compared to the average level pre-COVID. National-level estimates are also shown in the main paper in **fig. 2**.

[a] In South Africa, the analysis only included the KwaZulu-Natal Province and does not represent national-level disruptions.

Supplementary table 5. Country-specific factors promoting and disrupting health services during the pandemic

	**Daily new COVID-19 cases per million in 2020***	**Factors promoting continuity of health services during the pandemic**	**Other factors affecting health care use during the study period**
Chile	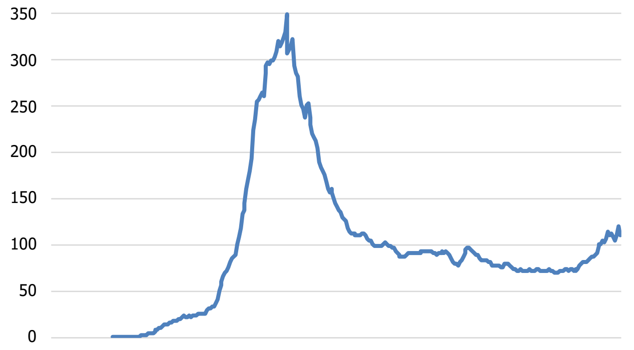	• Routine maternal health care is conducted exclusively by midwives. These services were able to continue as planned during the pandemic as midwives were not redeployed to COVID care.<br>• New online platform (*Saludable mente*) developed by the Ministry of Health to provide mental health care during the pandemic. [1]<br>• Home visits, telemedicine, and drug delivery implemented for higher risk populations including those with hypertension and diabetes.<br>• All ICU beds in public and private hospitals were centralized to better coordinate hospitalizations and referrals across the country.<br>• Most primary care facilities have separate emergency care units. These units were converted into *respiratory ERs* while non-respiratory care continued in separate areas.<br>• Pharmacies reported increased sales of some essential medicines including antihypertensives, pain killers, and birth control pills.	• Social unrest resulted in lower baseline utilization in November and December 2019 for some services.<br>• Many primary care personnel in Chile are aged over 60 and have chronic diseases. Many shifted to working from home because of their age and comorbidities.<br>• COVID and mental health effects on health workforce led to high absenteeism.
Ethiopia	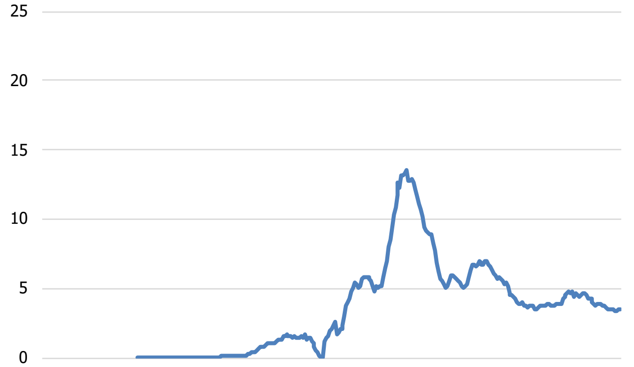	• Most of the country lives in rural areas that were not under lockdowns.<br>• No stay-at-home requirements. Other containment policies were declared but rarely enforced.<br>• National guidance disseminated: *Implementation Guide for Non-COVID-19 Essential Health Services in Ethiopia During COVID-19 Pandemic.* [2]<br>• Continuous monitoring of essential health services was made integral to the COVID response.<br>• Telephone consultations began for patient follow-up.<br>• Mass media campaign to encourage utilization of essential health care especially maternal, child health care and family planning services.<br>• Health workers received hazard pay.	• Conflicts in Tigray in late 2020 affected service delivery and dhis2 reporting in the region (region) was removed from the analysis.
Ghana	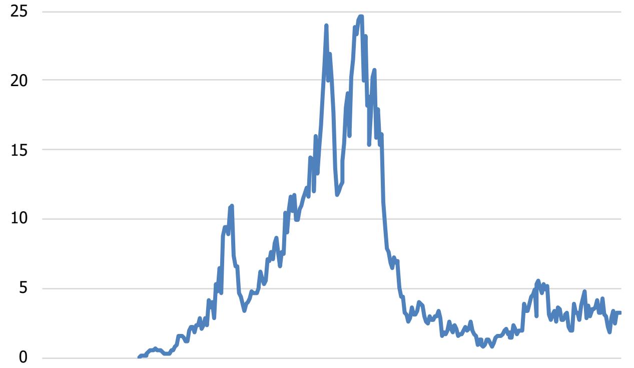	• Guidelines disseminated to ensure safe and quality RMNCH care. [3]<br>• Increased outreach and home visits for child vaccination.<br>• Strict time slots implemented for patient visits at Greater Accra regional hospital to allow physical distancing [3]	• Population advised to stay home unless the health need is critical.<br>• Specialist consultations made available only by appointment during the pandemic.

Haiti	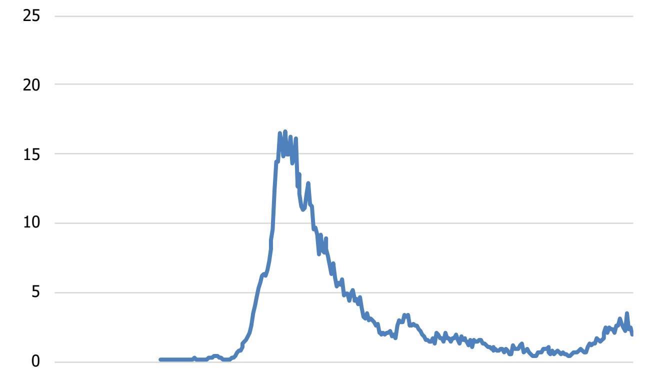	• Hospitals stopped providing general primary care but prioritized maternal and child health services, emergency care, HIV, TB, and oncology.	• Important social and political disturbances both before and during COVID and exacerbation in late 2020<br>• Most tertiary level hospitals postponed planned elective surgeries.
Lao PDR	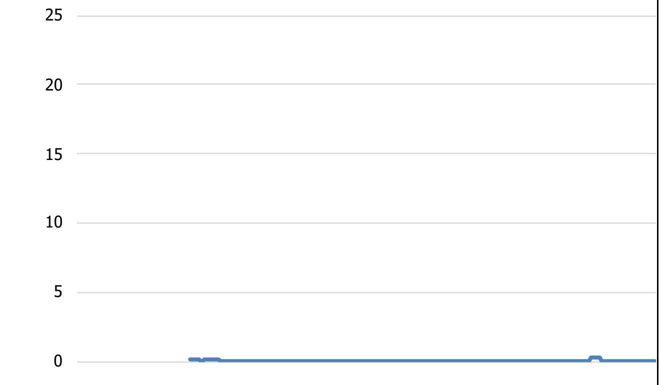	• National guidance disseminated on prioritization of RMNCAH services.<br>• Nation-wide training on infection control and safe provision of essential health services during the pandemic (screening, patient flow, zoning, safe transfer, cleaning and disinfection, PPE practice)<br>• Communication with public on the infection control effort in healthcare facilities<br>• Planning for surge capacity in hospitals considered maintaining capacity for routine services.<br>• Regular monitoring of essential health care utilization during the pandemic.	• Return of migrant workers from neighboring countries during the pandemic resulted in higher population of young adults seeking HIV/TB testing and reproductive health services
Mexico	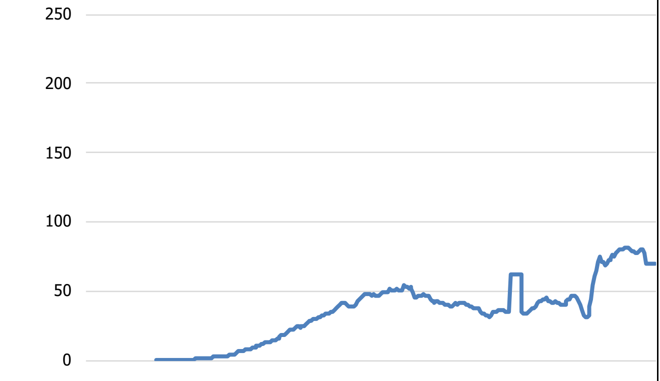	• Agreement signed with private hospitals to provide obstetric services and surgeries for IMSS patients from April 23 to May 23, 2020.<br>• Online refill and electronic prescriptions launched for patients with chronic diseases at the beginning of the pandemic.<br>• National vaccination days against measles, mumps, and rubella began in November 2020<br>• In April 2021, IMSS launched the National Strategy for Health Services Recovery to reorganize and resume services that had initially been suspended. This included weekend opening hours and the reconversion of some COVID facilities back to routine care.	• Most routine (non-urgent) health care appointments were canceled or postponed during the first months of the pandemic

Nepal	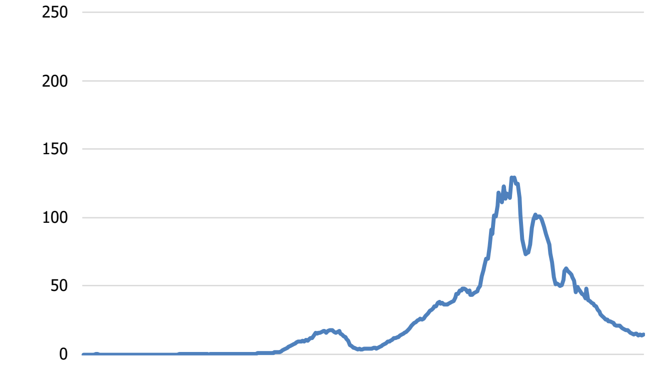	• Announcement of risk allowances for health workers. [4]<br>• Emergency guidelines formulated for maintaining SRMNCAH, HIV, TB, and Hepatitis C services and harm reduction programs for people who inject drugs during the pandemic. [5]<br>• Telephone helplines established for COVID and SRMNCAH services.<br>• Reproductive health and COVID-19 communication campaigns (flyers, radio and television PSAs).<br>• National measles, mumps and rubella vaccination campaign resumed in April 2020, along with all routine immunization, and rotavirus vaccine introduced in July 2020. [5]	• All health program budgets were frozen in the last quarter of fiscal year 2020 except for COVID-19 programming.<br>• Fear of discrimination by minorities may have led to further health care avoidance particularly following the incidence with Muslim Jamatis.<br>• Shortage of essential maternal and newborn health supplies including PPE. [5]
South Africa	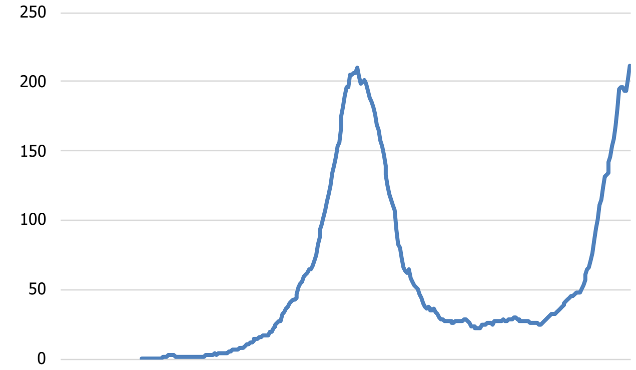	• Expansion of the Central Chronic Medicines Dispensing and Distribution (CCMDD) program prior to the national lockdown in late March 2020. The CCMDD, implemented in 2014, aims to increase and facilitate access to medicine for chronic disease patients (HIV, hypertension, and diabetes) via external pick-up points (PuPs). These PuPs include commercial outlets, pharmacy chains or community-based sites like churches.<br>• Increased use of telemedicine.<br>• Guidelines released on mental health interventions during the COVID-19 disaster [6]<br>• Telephone hotlines established to provide frontline workers with mental health care. [7]	• Complete ban on alcohol sales during the South African lockdowns led to fewer accidents and lower need for trauma care.<br>• Important efforts deployed in March 2020 to improve water provision and sanitation measures in high-density areas, informal settlements and rural areas leading to important reductions in diarrhea cases.
South Korea	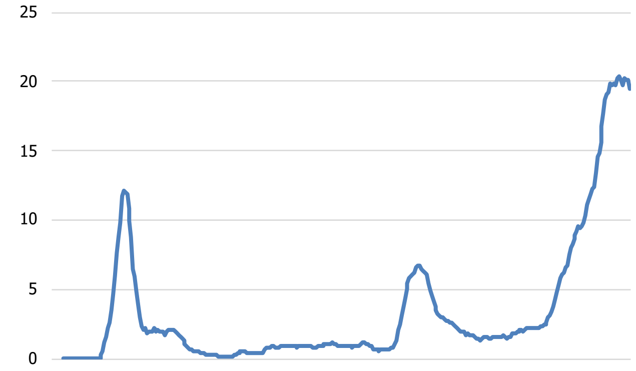	• A strong public health response system already in place prior to COVID, given Korea's experience handling the SARS outbreak of 2003, the novel influenza outbreak of 2009, and the MERSCoV epidemic of 2015.<br>• Creation of two parallel health systems at the beginning of the pandemic (a COVID system and a non-COVID system) to ensure continuity of non-COVID related needs. [8]<br>• Non-COVID-19 patients diverted through triage centers at the district or hospital level (designated as "system safety guaranteed hospitals" by the government). [8]<br>• Telemedicine temporarily allowed for established patients. [9]	• Experience with the 2015 MERS-CoV epidemic led the population to adopt extremely precautious behaviors leading to important reductions in non-COVID infectious illnesses.

Thailand	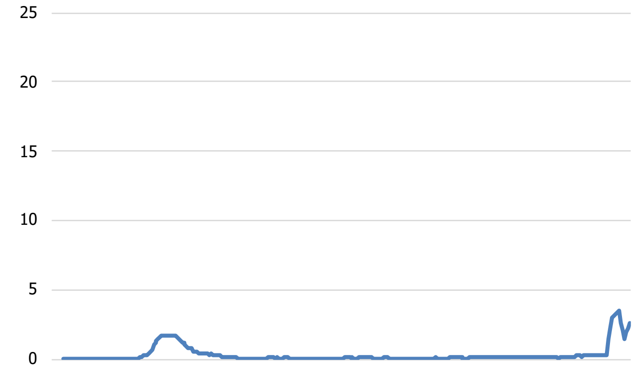	• Village health volunteer (VHC) allowed to dispense medications for stable chronic disease patients.<br>• Telemedicine and shipping prescribed drugs by mail for chronic disease patients. [10]<br>• Health workers offered hazard pay.<br>• Under Thailand's Universal Health Coverage people have no financial barriers to accessing obstetric services.	• Strict lockdown policies included outpatient healthcare facilities as restricted points of "mass-gathering".<br>• Public sector health workers expected to conduct disease control activities (investigating outbreaks and providing quarantine services) in addition to providing routine care.

* Smoothed daily new COVID-19 cases per million population. All graphs are from January 22, 2020 (date of the first reported case across the 10 countries) to December 31, 2020 (except in Nepal where the graph ends on January 13, 2021, to match the outcome (DHIS2) data). The y-axis differs by country.

Factors common across many countries (not included in table above): barriers imposed by lockdowns (e.g., curfews, stay-at-home orders, public transport closures), fear of contracting COVID at health facilities leading to reduced attendance, inability to pay for health care due to loss of employment or remuneration, converting health facilities into COVID centers and redeploying health workforce to COVID care. **SRMNCAH** is sexual, reproductive, maternal, neonatal, child and adolescent health. **PPE** is personal protective equipment. **PSA** is public service announcement.

1. Gobierno de Chile. Saludable mente. https://www.gob.cl/saludablemente/. 2021.
2. Ministry of Health of Ethiopia. Implementation guide for non-Covid-19 essential health services in Ethiopia during Covid-19 pandemic, 2020.
3. World Health Organization Regional Office for Africa. Easing COVID-19 impact on core health services in Ghana. RC70 e-Journal. 2020.
4. Aryal M. Frontline health workers deprived of promised risk allowances. The Kathmandu Post. 2021.
5. Government of Nepal Ministry of Health and Population. Reponding to COVID-19, Health sector preparedness, response and lessons learnt. Kathmandu, Nepal, 2021.
6. Republic of South Africa. Guidelines on mental health interventions during the COVID-19 disaster., 2020.
7. Kola L, Kohrt BA, Hanlon C, et al. COVID-19 mental health impact and responses in low-income and middle-income countries: reimagining global mental health. *The Lancet Psychiatry* 2021.
8. Oh J, Lee JK, Schwarz D, Ratcliffe HL, Markuns JF, Hirschhorn LR. National Response to COVID-19 in the Republic of Korea and Lessons Learned for Other Countries. *Health Syst Reform* 2020; **6**(1): e1753464.
9. Haldane V, De Foo C, Abdalla SM, et al. Health systems resilience in managing the COVID-19 pandemic: lessons from 28 countries. *Nat Med* 2021; **27**(6): 964-80.
10. Songsermpong S, Bunluesin S, Khomgongsuwan P, et al. Innovations to Sustain Non-Communicable Disease Services in the Context of COVID-19: Report from Pakkred District, Nonthaburi Province, Thailand. *Glob Heart* 2021; **16**(1): 44.

Supplementary table 6. Characteristics of countries included

Country/Region	Income group	Population millions[a]	Current health expenditure (% of GDP)[a]	Domestic public health expenditure (% of health expenditure)[a]	Domestic private health expenditure (% of health expenditure)[a]	UHC Service coverage index[c]	Out-of-pocket health expenses % of current health expenditure [a]
**East and South Asia**							
Lao PDR	LMI	7.3	2.2	38.7	48.9	51.0	48.5
Nepal	LMI	29.1	5.8	25.0	65.9	48.0	50.8
South Korea	HI	51.8	7.6	58.5	41.5	86.0	32.5
Thailand	UMI	69.8	3.8	76.3	23.4	80.0	11.0
**Latin America and Caribbean**							
Chile	HI	19.2	9.1	50.8	49.2	70.0	33.2
Haiti	LI	11.4	7.7	12.0	48.8	49.0	43.6
Mexico	UMI	128.9	5.4	50.0	49.9	76.0	42.1
**Sub-Saharan Africa**							
Ethiopia	LI	115.0	3.3	23.3	40.7	39.0	35.5
Ghana	LMI	31.1	3.5	38.9	48.7	47.0	37.7
South Africa	UMI	11.5 [b]	8.3	54.0	44.1	69.0	7.7

LI lower income **LMI** lower middle income **UMI** upper middle-income **HI** high income

a. The World Bank, World Development Indicators (2020)
b. Population is for KwaZulu-Natal Province only. Other indicators are for South Africa as a whole. Source for KwaZulu-Natal population: Statistics South Africa. (2020). (rep.). Statistical release: Mid-year population estimates. Retrieved from http://www.statssa.gov.za/publications/P0302/P03022020.pdf
c. World Health Organization, Global Health Observatory Data Reposit

Supplementary table 7. Health conditions addressed by the Sustainable Development Goals (SDGs) and covered in the analysis

**SDG target**	**Health condition**	**Covered in the analysis**
3.1	Maternal health	X
3.2	Newborn health	X
3.2	Child health	X
3.3	HIV/AIDS	X
3.3	Tuberculosis	X
3.3	Malaria	X
3.3	Hepatitis B	X
3.3	Neglected tropical diseases	
3.4	Cardiovascular diseases	X
3.4	Cancer	X
3.4	Diabetes	X
3.4	Chronic respiratory disease	
3.4	Mental health	X
3.5	Substance abuse	
3.6, 16.1	Injuries from road traffic accidents and violence	X
3.7	Sexual and reproductive health	X

Supplementary table 8. Sensitivity analysis using different reporting thresholds for inclusion during data cleaning

Ethiopia

Health service	Month threshold	% visits excluded	RD Covid	p-value	RD Q4 2020	p-value
Deliveries	12	-0.4%	-505	0.33	323	0.65
	15	-0.7%	-448	0.35	386	0.60
	18	-1.6%	-282	0.47	599	0.48
	24	-16.6%	-70	0.81	651	0.45
Inpatient admissions	12	-4.6%	-2945	0.04	-1509	0.07
	15	-6.0%	-3190	0.03	-1555	0.04
	18	-9.9%	-2822	0.04	-1260	0.03
	24	-44.8%	-1692	0.05	-561	0.06
Outpatient visits	12	-2.1%	-179804	0.06	-55277	0.17
	15	-2.8%	-175660	0.06	-52044	0.19
	18	-3.4%	-170335	0.06	-46175	0.22
	24	-19.5%	-145618	0.08	-35442	0.31
Pentavalent vaccines	12	-0.8%	-1038	0.22	-232	0.71
	15	-1.0%	-990	0.21	-184	0.78
	18	-1.4%	-919	0.24	-93	0.89
	24	-10.9%	-893	0.20	241	0.66

Haiti

Health service	Month threshold	% visits excluded	RD Covid	p-value	RD Q4 2020	p-value
Deliveries	12	-2.1%	-306	0.01	-151	0.04
	15	-2.5%	-307	0.01	-153	0.05
	18	-5.6%	-309	0.01	-152	0.04
	24	-42.1%	-173	0.00	-54	0.06
Diabetes visits	12	-7.7%	-311	0.04	-133	0.01
	15	-9.9%	-306	0.05	-131	0.01
	18	-17.8%	-246	0.01	-101	0.03
	24	-85.0%	-39	0.05	-3	0.70
Outpatient visits	12	-5.7%	-21829	0.09	-2812	0.17
	15	-6.1%	-21742	0.09	-2639	0.18
	18	-7.9%	-21040	0.09	-2947	0.26
	24	-32.7%	-9368	0.01	2318	0.47

Lao PDR

Health service	Month threshold	% visits excluded	RD Covid	p-value	RD Q4 2020	p-value
Deliveries	12	-1.2%	-23	0.04	74	0.01
	15	-2.8%	-23	0.04	65	0.00
	18	-4.7%	-23	0.05	65	0.00
	24	-16.6%	-23	0.07	57	0.00

Health service	Month threshold	% visits excluded	RD Covid	p-value	RD Q4 2020	p-value
Diabetes visits	12	-2.0%	4	0.83	-14	0.68
	15	-2.6%	3	0.90	-12	0.74
	18	-4.0%	5	0.80	-8	0.81
	24	-34.8%	-18	0.45	-18	0.67
Inpatient admissions	12	-1.5%	-1064	0.00	686	0.16
	15	-4.8%	-1082	0.00	698	0.16
	18	-5.3%	-1070	0.00	706	0.16
	24	-10.0%	-1054	0.00	684	0.17
Outpatient visits	12	-1.3%	-9040	0.00	-3594	0.01
	15	-1.7%	-9037	0.00	-3541	0.01
	18	-1.9%	-8960	0.00	-3438	0.01
	24	-5.9%	-8787	0.00	-3913	0.00
Pentavalent vaccines	12	-1.0%	-159	0.00	85	0.05
	15	-2.9%	-154	0.00	82	0.05
	18	-5.8%	-145	0.00	80	0.04
	24	-32.8%	-65	0.00	56	0.03

Nepal

Health service	Month threshold	% visits excluded	RD Covid	p-value	RD Q4 2020	p-value
Deliveries	12	-0.3%	-48	0.00	-92	0.02
	15	-0.3%	-49	0.00	-92	0.02
	18	-1.0%	-50	0.00	-92	0.02
	24	-11.1%	-44	0.00	-91	0.02
Diabetes visits	12	-1.5%	-181	0.00	-186	0.00
	15	-2.2%	-183	0.00	-188	0.00
	18	-3.9%	-181	0.00	-189	0.00
	24	-12.1%	-175	0.00	-186	0.00
Inpatient admissions	12	-9.7%	-197	0.04	-300	0.06
	15	-11.1%	-194	0.04	-301	0.06
	18	-15.9%	-195	0.04	-279	0.08
	24	-36.6%	-192	0.03	-213	0.16
Outpatient visits	12	-4.1%	-3045	0.00	-3913	0.00
	15	-4.1%	-3115	0.00	-3915	0.00
	18	-4.1%	-3114	0.00	-3917	0.00
	24	-5.6%	-3077	0.00	-3905	0.00
Pentavalent vaccines	12	-0.1%	-284	0.00	64	0.01
	15	-0.2%	-284	0.00	64	0.01
	18	-0.2%	-284	0.00	64	0.01
	24	-46.2%	-145	0.00	12	0.32

South Africa

Health service	Month threshold	% visits excluded	RD Covid	p-value	RD Q4 2020	p-value
Deliveries	12	-0.4%	-224.4	0.01	-124.5	0.04
	15	-0.5%	-222.5	0.02	-123.1	0.05

	18	-0.5%	-222.4	0.02	-123.0	0.05
	24	-2.3%	-203.8	0.01	-94.4	0.03
Diabetes visits	12	-1.7%	-2038.7	0.08	-2841.5	0.07
	15	-1.8%	-2033.1	0.08	-2820.1	0.07
	18	-1.9%	-2028.8	0.08	-2819.3	0.07
	24	-4.4%	-1924.8	0.08	-2784.5	0.07
Inpatient admissions	12	-0.0%	-1440.6	0.03	-653.4	0.06
	15	-0.0%	-1440.6	0.03	-653.4	0.06
	18	-0.01%	-1440.4	0.03	-653.2	0.06
	24	-0.03%	-1438.9	0.03	-651.4	0.06
Outpatient visits	12	0.0%	-11955.4	0.07	-7984.6	0.04
	15	0.0%	-11955.4	0.07	-7984.6	0.04
	18	0.0%	-11955.4	0.07	-7984.6	0.04
	24	0.0%	-11954.4	0.07	-7981.6	0.04
Pentavalent vaccines	12	-0.4%	-246.2	0.16	-26.8	0.36
	15	-0.6%	-240.0	0.17	-22.5	0.39
	18	-0.6%	-240.0	0.17	-22.5	0.39
	24	-2.9%	-215.4	0.17	-14.8	0.59

RD Covid is the risk difference for the average level change when the pandemic is declared. RD Q4 2020 is the remaining level change in the last quarter of 2020. Results using the 15-month reporting threshold for inclusion are those shown in the supplementary material 5 and the main analysis. Chile is not included in this sensitivity analysis as there were very few missing data and the complete case analysis had no impact on results. Ghana, Mexico, South Korea and Thailand are not included because the data were obtained at regional levels and data cleaning was performed by data custodians.

Supplementary table 9. Number of health care visits included in the datasets from January 2019 to December 2020 in six countries, before and after data cleaning

Variable	Total health care visits in the raw data	Total health care visits in the final data	Δ
**Chile**			
Antenatal care visits	304,758	304,284	0%
BCG	395,596	395,596	0%
Breast cancer screening	1,794,662	1,794,662	0%
Caesarean sections	120,768	120,768	0%
Deliveries	253,240	253,240	0%
Diabetes visits	137,343	134,237	-2%
Emergency room visits	27,899,424	27,885,976	0%
Family planning	2,867,267	2,865,226	0%
Hypertension visits	250,633	246,293	-2%
Inpatient admissions	2,966,985	2,966,985	0%
Measles	402,750	402,750	0%
Mental health care	6,679,714	6,667,139	0%
Pentavalent	398,894	398,894	0%
Postnatal care visits	171,461	171,360	0%
Pneumococcal	406,778	406,778	0%
Pneumonia	35,527	35,527	0%
Road traffic accidents	183,953	182,343	-1%
Surgeries	1,770,064	1,768,585	0%
		**Average:**	**0%**
**Haiti**			
Antenatal care visits	1,465,122	1,403,546	-4%
Deliveries	226,883	221,169	-3%
Diabetes visits	129,945	117,081	-10%
Family planning	9,856,483	9,406,444	-5%
Hypertension visits	466,303	440,090	-6%
Outpatient visits	16,845,210	15,820,037	-6%
Postnatal care visits	258,854	241,132	-7%
Fully vaccinated by 1	265,430	232,209	-13%
		**Average:**	**-7%**
**Lao PDR**			
Antenatal care visits	321,650	316,144	-2%
BCG	282,942	263,755	-7%
Caesarean sections	22,362	21,769	-3%
Deliveries	221,011	214,754	-3%
Diabetes visits	111,076	108,137	-3%
Family planning	9,778,392	9,657,448	-1%
Hypertension visits	297,585	288,867	-3%
Inpatient admissions	1,319,616	1,256,878	-5%
Outpatient visits	12,132,465	11,929,433	-2%
Pentavalent	278,559	270,512	-3%
Postnatal care visits	304,924	295,279	-3%
Pneumococcal	276,829	266,734	-4%
Road traffic accidents	207,259	190,464	-8%
		**Average:**	**-3%**
**Nepal**			
Antenatal care visits	1,350,440	1,338,218	-1%
BCG	1,106,951	1,102,239	0%
Caesarean sections	162,179	160,445	-1%
Deliveries	755,702	753,255	0%
Diabetes visits	569,412	557,099	-2%
Diarrhea	725,826	718,567	-1%
Emergency room visits	4,176,263	3,950,569	-5%
Family planning	13,316,456	13,152,574	-1%
HIV tests	1,622,636	1,584,622	-2%
Hypertension visits	1,311,386	1,189,441	-9%
Inpatient admissions	973,248	865,527	-11%
Measles	1,900,831	1,882,673	-1%
Outpatient visits	34,241,012	32,827,482	-4%
Pentavalent	1,028,242	1,026,692	0%

Variable	Total health care visits in the raw data	Total health care visits in the final data	Δ
**Ethiopia**			
Antenatal care visits	7,194,426	7,115,600	-1%
People on ART	10,961,491	10,882,359	-1%
BCG	6,429,882	6,360,562	-1%
Caesarean sections	289,803	278,382	-4%
Deliveries	4,264,745	4,233,359	-1%
Diarrhea	1,258,695	1,131,196	-10%
Emergency room visits	6,892,143	6,288,737	-9%
Family planning	27,764,602	27,385,784	-1%
Inpatient admissions	2,676,455	2,515,272	-6%
Measles	5,946,868	5,853,584	-2%
Outpatient visits	200,597,712	195,061,328	-3%
Pentavalent	6,304,932	6,242,822	-1%
Postnatal care visits	5,566,428	5,501,365	-1%
Pneumococcal	6,258,321	6,201,392	-1%
Pneumonia	4,204,742	4,111,499	-2%
Road traffic accidents	494,857	423,833	-14%
Rotavirus	6,219,108	6,169,407	-1%
Malnutrition	135,617,552	133,401,752	-2%
Fully vaccinated by 1	5,667,403	5,568,665	-2%
		**Average:**	**-3%**
**South Africa**			
Antenatal care visits	458,017	456,105	0%
People on ART	34,848,184	34,821,584	0%
BCG	393,271	385,619	-2%
Cervical cancer screening	301,929	290,830	-4%
Caesarean sections	147,162	144,850	-2%
Deliveries	285,098	283,665	-1%
Diabetes visits	2,163,529	2,124,958	-2%
Diarrhea	15,563	15,406	-1%
Inpatient admissions	1,347,080	1,346,988	0%
Measles	428,689	423,373	-1%
Outpatient visits	9,448,153	9,448,153	0%
Pentavalent	424,439	421,784	-1%
Postnatal care visits	324,750	323,799	0%
Pneumococcal	467,888	464,272	-1%
Pneumonia	63,718	59,949	-6%
Road traffic accidents	89,405	89,238	0%
Rotavirus	417,018	413,308	-1%
TB detection	76,302	75,070	-2%
TB screening	43,722,336	43,522,312	0%
TB treatment	75,248	74,798	-1%
Trauma admissions	58,396	57,826	-1%
Fully vaccinated by 1	467,062	463,012	-1%
		**Average:**	**-1%**

Postnatal care visits	244,999	238,436	-3%
Pneumococcal	988,954	982,775	-1%
Pneumonia	229,300	225,630	-2%
TB detection	39,478	34,141	-14%
		**Average:**	**-3%**

Δ is the % difference in health care visits over 24 months between the raw and cleaned datasets.

Supplementary tables 10-19. Estimates from segmented regression analyses for the impact of the pandemic on health service levels in 2020

Supplementary table 10

Chile	Number of regions:	16				Pandemic impact								Potential resumption period			
**Health service**	**Average over the pre-Covid period**	**Avg monthly change before the pandemic (secular trend β1)**				**Level change due to COVID-19 (change in level β2)**				**Avg monthly change during the pandemic (change in slope β3)**				**Remaining level change in the last quarter of 2020 (remaining change in level β4)**			
		Coeff	LCL	UCL	p-value	Coeff	LCL	UCL	p-value	Coeff	LCL	UCL	p-value	Coeff	LCL	UCL	p-value
Family planning visits	8,951	-127	-205	-49	0.004	-7,791	-13,254	-2,329	0.008	647	190	1,103	0.009	-2,800	-4,589	-1,011	0.005
Antenatal care visits	815	-9	-17	-2	0.015	-59	-175	58	0.301	12	-6	29	0.184	-7	-43	30	0.691
Deliveries	665	-6	-14	3	0.159	8	-15	30	0.478	9	0	17	0.049	49	2	96	0.041
C-sections	311	-1	-4	2	0.544	12	-8	32	0.214	4	0	8	0.063	29	0	58	0.053
Postnatal care visits	459	-6	-12	0	0.048	-50	-107	7	0.082	13	8	18	0.000	26	-2	54	0.067
Pneumonia	121	-2	-5	1	0.171	-95	-205	16	0.087	-29	-61	3	0.072	-100	-183	-17	0.021
BCG vaccine	1,045	-6	-10	-1	0.021	-42	-94	10	0.104	8	5	11	0.000	-3	-46	39	0.877
Pentavalent vaccine	1,064	-5	-11	0	0.054	-210	-485	64	0.123	38	-10	86	0.111	-16	-41	10	0.205
Measles vaccine	1,088	-2	-4	0	0.053	-187	-480	105	0.191	18	-21	57	0.337	-100	-196	-4	0.043
Pneumococcal vaccine	1,098	-2	-3	0	0.013	-178	-469	113	0.211	15	-22	52	0.394	-103	-211	5	0.059
Inpatient admissions	8,315	-33	-70	3	0.070	-3,896	-7,629	-162	0.042	452	-5	910	0.052	-195	-548	157	0.254
Emergency room visits	81,814	-847	-1,721	28	0.057	-48,078	-85,721	-10,436	0.016	2,445	657	4,232	0.011	-30,410	-52,309	-8,512	0.010
Surgeries	5,204	-49	-87	-10	0.017	-3,248	-5,698	-799	0.013	310	26	595	0.035	-444	-785	-104	0.014
Road traffic accidents	521	-2	-10	5	0.544	-347	-754	59	0.088	52	-51	155	0.297	-105	-210	-1	0.048
Diabetes visits	407	-9	-16	-1	0.024	-326	-611	-42	0.027	45	1	89	0.044	21	-23	65	0.322
Hypertension visits	750	-17	-29	-5	0.010	-614	-1,206	-21	0.043	74	6	141	0.034	-19	-83	45	0.534
Mental health care	20,812	-285	-539	-30	0.031	-17,433	-32,108	-2,759	0.023	1,079	236	1,922	0.016	-7,132	-13,008	-1,255	0.021
Breast cancer screening	5,636	-6	-58	46	0.813	-5,417	-9,721	-1,113	0.017	295	-75	665	0.110	-775	-1,432	-117	0.024

The pre-pandemic period is January 1, 2019, to March 31, 2020 (15 months). The pandemic impact period is April 1 to September 30, 2020 (6 months) and the potential resumption period is October 1 to December 31, 2020 (3 months).

Supplementary table 11.

Ethiopia	Number of regions: 10					Pandemic impact								Potential resumption period			
**Health service**	**Average over the pre-Covid period**	**Avg monthly change before the pandemic (secular trend $\beta1$)**				**Level change due to COVID-19 (change in level $\beta2$)**				**Avg monthly change during the pandemic (change in slope $\beta3$)**				**Remaining level change in the last quarter of 2020 (remaining change in level $\beta4$)**			
		Coeff	LCL	UCL	p-value	Coeff	LCL	UCL	p-value	Coeff	LCL	UCL	p-value	Coeff	LCL	UCL	p-value
Family planning visits	108,186	475	-316	1,266	0.203	-7,326	-16,185	1,532	0.093	2,657	-1,103	6,417	0.142	-290	-6,324	5,743	0.914
Antenatal care visits	28,381	38	-17	92	0.149	-3,904	-8,636	829	0.094	907	-246	2,060	0.107	32	-977	1,041	0.944
Deliveries	16,666	38	-9	84	0.099	-448	-1,485	590	0.349	126	-267	519	0.482	386	-1,258	2,031	0.603
Caesarean sections	1,073	9	0	18	0.056	-11	-76	53	0.692	3	-10	16	0.653	-32	-179	115	0.627
Postnatal care visits	21,673	129	5	253	0.043	-538	-1,532	457	0.248	88	-353	528	0.659	-49	-2,199	2,101	0.959
Diarrhea	4,622	71	-32	174	0.152	-747	-1,349	-146	0.021	8	-78	94	0.830	-259	-953	436	0.415
Pneumonia	16,945	397	-113	907	0.110	-3,842	-8,017	334	0.067	-648	-1,310	13	0.054	-6,087	-12,257	83	0.052
Malnutrition	511,608	7,179	-6,136	20,493	0.249	-30,398	-71,730	10,935	0.128	5,789	-994	12,572	0.085	42,081	-15,735	99,896	0.132
Fully vaccinated by 1	21,981	74	-17	165	0.098	-199	-860	461	0.506	132	-140	404	0.295	718	-1,256	2,692	0.426
BCG vaccine	25,104	-6	-68	56	0.831	-1,284	-3,698	1,129	0.255	688	-319	1,695	0.154	2,206	-1,256	5,667	0.180
Pentavalent vaccine	24,702	112	11	212	0.034	-990	-2,679	700	0.214	225	-246	695	0.303	-184	-1,646	1,278	0.779
Measles vaccine	23,131	91	-4	186	0.058	-390	-1,444	663	0.418	126	-217	470	0.422	316	-1,421	2,052	0.686
Pneumococcal vaccine	24,539	108	9	206	0.036	-973	-2,746	799	0.241	230	-261	720	0.311	-170	-1,637	1,296	0.796
Rotavirus vaccine	24,336	100	-2	201	0.053	-524	-1,882	834	0.400	161	-265	587	0.409	-275	-1,491	941	0.616
People on ART	41,577	45	-21	110	0.156	-141	-1,098	816	0.742	25	-259	309	0.844	399	-376	1,174	0.269
Outpatient visits	743,791	10,233	-1,951	22,416	0.089	-175,660	-362,578	11,259	0.062	14,238	-1,749	30,226	0.074	-52,044	-136,089	32,001	0.191
Emergency room visits	23,165	386	25	748	0.039	-9,549	-18,072	-1,026	0.032	613	-276	1,502	0.150	-4,558	-8,183	-933	0.020
Inpatient admissions	10,042	79	1	158	0.048	-3,190	-6,080	-299	0.034	179	-2	361	0.052	-1,555	-3,058	-51	0.044
Road traffic accidents	1,728	20	-9	49	0.150	-345	-745	56	0.082	49	-1	98	0.054	-142	-389	105	0.221

In Ethiopia, the pre-pandemic period is Tirr 2011 to Yekatit 2012 (January 9, 2019, to March 9, 2020) (14 months). The pandemic impact period is Megabit to Nashi 2012 (March 10 to September 5, 2020) (6 months) and the potential resumption period is Meskerem to Tahsas 2013 (September 6, 2020, to January 9, 2021) (4 months).

Supplementary table 12.

Ghana	**Number of units:**	**16**				**Pandemic impact**								**Potential resumption period**			
**Health service**	**Average over the pre-Covid period**	**Avg monthly change before the pandemic (secular trend $\beta 1$)**				**Level change due to COVID-19 (change in level $\beta 2$)**				**Avg monthly change during the pandemic (change in slope $\beta 3$)**				**Remaining level change in the last quarter of 2020 (remaining change in level $\beta 4$)**			
		Coeff	LCL	UCL	p-value	Coeff	LCL	UCL	p-value	Coeff	LCL	UCL	p-value	Coeff	LCL	UCL	p-value
Family planning	13,997	-435	-935	65	0.083	-103	-1,827	1,621	0.900	555	-268	1,378	0.170	3,049	-586	6,684	0.094
Antenatal care visits	24,845	62	21	102	0.005	-2,142	-4,157	-128	0.039	309	-24	641	0.066	1,677	424	2,930	0.012
Deliveries	3,829	19	-31	70	0.429	265	-235	765	0.275	-104	-161	-47	0.002	-52	-866	762	0.894
Caesarean sections	692	-1	-3	2	0.644	124	46	202	0.004	-16	-27	-4	0.012	116	35	197	0.008
Postnatal care visits	3,907	-8	-18	2	0.129	608	359	856	0.000	-104	-165	-43	0.003	397	144	651	0.005
Diarrhea	3,175	-28	-41	-14	0.001	-803	-1,169	-438	0.000	-25	-68	19	0.250	-4	-275	266	0.973
Pneumonia	817	9	-4	21	0.156	-520	-830	-210	0.003	-40	-58	-22	0.000	-108	-310	95	0.272
Fully vaccinated by 1	5,119	-2	-25	21	0.839	7	-455	468	0.977	-172	-331	-13	0.036	-340	-754	74	0.100
BCG vaccine	6,013	-25	-52	2	0.069	222	-55	498	0.108	5	-71	81	0.886	788	287	1,290	0.005
Pentavalent vaccine	6,025	-14	-46	18	0.360	311	118	504	0.004	-30	-89	29	0.294	510	146	874	0.009
Measles vaccine	11,228	-19	-80	41	0.504	-250	-633	134	0.184	-15	-151	122	0.821	479	-274	1,232	0.194
Pneumococcal vaccine	6,157	-10	-35	15	0.414	-451	-795	-106	0.014	138	25	250	0.020	206	-102	514	0.173
Rotavirus vaccine	11,729	-25	-89	38	0.405	298	-48	644	0.086	-45	-190	101	0.523	398	-237	1,033	0.200
Outpatient visits	169,603	647	-40	1,334	0.063	-33,653	-50,874	-16,432	0.001	-2,220	-3,632	-808	0.005	-11,270	-21,973	-566	0.040
Inpatient admissions	9,672	53	16	89	0.008	-1,465	-2,018	-913	0.000	-109	-225	7	0.064	-263	-785	260	0.299
Road traffic accidents	545	4	-4	11	0.321	-105	-301	90	0.268	28	0	55	0.051	144	26	263	0.021
Diabetes visits	932	-10	-21	2	0.087	-61	-287	165	0.572	35	-18	88	0.175	187	-54	427	0.119
Hypertension visits	3,249	-18	-60	25	0.391	-226	-917	465	0.495	49	-46	145	0.286	53	-870	977	0.903
Malaria visits	33,105	20	-122	162	0.765	-2,876	-4,984	-768	0.011	-1,318	-1,977	-658	0.001	-1,432	-4,266	1,401	0.297
TB detection	154	0	-1	1	0.793	-47	-90	-4	0.036	0	-3	4	0.762	-23	-52	5	0.102

The pre-pandemic period is January 1, 2019, to March 31, 2020 (15 months). The pandemic impact period is April 1 to September 30, 2020 (6 months) and the potential resumption period is October 1 to December 31, 2020 (3 months).

Supplementary table 13.

Haiti	Number of départements: 10					Pandemic impact								Potential resumption period			
**Health service**	**Average over the pre-Covid period**	**Avg monthly change before the pandemic (secular trend $\beta1$)**				**Level change due to COVID-19 (change in level $\beta2$)**				**Avg monthly change during the pandemic (change in slope $\beta3$)**				**Remaining level change in the last quarter of 2020 (remaining change in level $\beta4$)**			
		Coeff	LCL	UCL	p-value	Coeff	LCL	UCL	p-value	Coeff	LCL	UCL	p-value	Coeff	LCL	UCL	p-value
Outpatient visits	67,168	230	-328	787	0.370	-21,742	-48,206	4,722	0.095	3,151	-1,318	7,621	0.143	-2,639	-6,777	1,498	0.180
Family planning	38,746	335	-196	866	0.183	-5,481	-16,359	5,397	0.279	693	-1,344	2,730	0.456	-642	-3,415	2,131	0.608
Antenatal care visits	6,002	-90	-164	-16	0.024	-1,959	-4,026	108	0.060	450	18	883	0.043	408	-107	923	0.105
Deliveries	987	-2	-7	4	0.536	-307	-517	-97	0.010	18	-14	49	0.233	-153	-301	-4	0.045
Postnatal care visits	1,063	3	-3	9	0.306	-376	-655	-96	0.015	42	9	75	0.019	-159	-406	88	0.176
Fully vaccinated by age 1	965	18	7	29	0.006	-666	-1,199	-132	0.021	125	37	213	0.012	-196	-343	-50	0.015
Diabetes visits	494	8	-1	17	0.067	-306	-611	-1	0.049	42	-16	99	0.136	-131	-222	-40	0.010
Hypertension visits	1,906	28	-4	60	0.081	-1,271	-2,346	-196	0.026	151	-15	316	0.069	-394	-802	15	0.057

The pre-pandemic period is January 1, 2019, to March 31, 2020 (15 months). The pandemic impact period is April 1 to September 30, 2020 (6 months) and the potential resumption period is October 1 to December 31, 2020 (3 months).

Supplementary table 14.

Lao, PDR	Number of provinces 18					Pandemic impact								Potential resumption period			
**Health service**	**Average over the pre-Covid period**	**Avg monthly change before the pandemic (secular trend $\beta 1$)**				**Level change due to COVID-19 (change in level $\beta 2$)**				**Avg monthly change during the pandemic (change in slope $\beta 3$)**				**Remaining level change in the last quarter of 2020 (remaining change in level $\beta 4$)**			
		Coeff	LCL	UCL	p-value	Coeff	LCL	UCL	p-value	Coeff	LCL	UCL	p-value	Coeff	LCL	UCL	p-value
Outpatient visits	27,948	251	159	343	0.000	-9,037	-11,510	-6,563	0.000	1,160	525	1,795	0.001	-3,541	-6,033	-1,050	0.008
Inpatient admissions	2,941	-25	-52	1	0.061	-1,082	-1,658	-505	0.001	314	39	588	0.028	698	-297	1,693	0.156
Family planning	22,718	6	-217	228	0.958	-1,847	-4,825	1,130	0.207	-50	-274	174	0.643	-305	-2,601	1,990	0.781
Antenatal care visits	717	-1	-2	1	0.532	-72	-109	-34	0.001	36	24	48	0.000	5	-45	55	0.828
Deliveries	497	0	-2	1	0.971	-23	-45	-1	0.040	16	9	23	0.000	65	25	105	0.004
Caesarean sections	49	0	-1	0	0.856	3	-6	13	0.469	2	0	4	0.025	16	-3	35	0.093
Postnatal care visits	687	0	-4	4	0.973	-59	-104	-14	0.013	20	0	39	0.047	43	-23	109	0.183
BCG vaccine	611	1	-2	5	0.378	-169	-239	-98	0.000	52	25	80	0.001	83	7	160	0.035
Pentavalent vaccine	638	-1	-4	1	0.324	-154	-227	-81	0.000	36	15	57	0.002	82	0	163	0.049
Pneumococcal vaccine	628	-1	-4	1	0.314	-155	-233	-77	0.001	37	14	59	0.003	75	-6	156	0.068
Diabetes visits	238	5	-2	12	0.143	3	-40	46	0.899	1	-8	10	0.823	-12	-83	60	0.735
Hypertension visits	662	8	4	12	0.000	-76	-143	-9	0.029	11	-3	26	0.125	-59	-140	21	0.138
Road traffic accidents	458	1	-2	4	0.643	-188	-321	-55	0.009	38	9	68	0.014	23	-23	69	0.306

The pre-pandemic period is January 1, 2019, to March 31, 2020 (15 months). The pandemic impact period is April 1 to September 30, 2020 (6 months) and the potential resumption period is October 1 to December 31, 2020 (3 months). PDR, People's Democratic Republic.

Supplementary table 15.

Mexico, IMSS		Number of delegations		35				Pandemic impact						Potential resumption period		
**Health service**	**Average over the pre-Covid period**	**Avg monthly change before the pandemic (secular trend $\beta 1$)**				**Level change due to COVID-19 (change in level $\beta 2$)**				**Avg monthly change during the pandemic (change in slope $\beta 3$)**				**Remaining level change in the last quarter of 2020 (remaining change in level $\beta 4$)**			
		Coeff	LCL	UCL	p-value	Coeff	LCL	UCL	p-value	Coeff	LCL	UCL	p-value	Coeff	LCL	UCL	p-value
Family planning	1,136	-22	-28	-15	0.000	-593	-737	-449	0.000	16	2	29	0.023	-310	-423	-197	0.000
Antenatal care visits	7,697	-49	-71	-28	0.000	-2,186	-2,698	-1,674	0.000	26	-29	82	0.344	-2,018	-2,630	-1,406	0.000
Deliveries	492	-3	-5	0	0.059	-11	-27	6	0.203	-22	-34	-10	0.001	-130	-198	-61	0.001
Caesarean sections	410	-2	-4	0	0.062	-22	-39	-4	0.015	-10	-18	-2	0.018	-65	-109	-20	0.005
Diarrhea	234	-15	-19	-12	0.000	-147	-175	-120	0.000	18	11	25	0.000	49	18	79	0.003
Pneumonia	6	0	-1	0	0.000	-1	-2	0	0.033	0	0	0	0.118	-1	-2	0	0.034
Malnutrition	50	-1	-1	0	0.000	-28	-36	-21	0.000	-2	-2	-1	0.000	-26	-33	-19	0.000
BCG vaccine	541	2	-3	8	0.413	-516	-665	-368	0.000	75	53	97	0.000	-618	-812	-424	0.000
Pentavalent vaccine	1,244	-48	-63	-33	0.000	-538	-675	-401	0.000	39	20	58	0.000	-160	-252	-68	0.001
Measles vaccine	188	-17	-23	-10	0.000	3	-61	67	0.919	40	25	55	0.000	326	214	438	0.000
Outpatient	242,718	-304	-499	-109	0.003	-98,295	-121,759	-74,831	0.000	-1,840	-2,837	-843	0.001	-81,191	-100,749	-61,633	0.000
Pneumococcal	128	-2	-4	0	0.014	-70	-95	-46	0.000	11	6	16	0.000	-5	-25	15	0.623
Rotavirus	446	-3	-7	1	0.127	-169	-223	-116	0.000	24	14	35	0.000	-53	-105	-2	0.041
Diabetes visits	36,531	92	-105	288	0.349	-9,585	-11,948	-7,222	0.000	-1,112	-1,678	-546	0.000	-11,960	-15,948	-7,973	0.000
Hypertension visits	39,199	-491	-748	-234	0.000	-11,095	-13,780	-8,410	0.000	655	-1	1,312	0.050	-1,608	-5,014	1,797	0.344
People on ART	1,321	-11	-29	7	0.233	-29	-149	91	0.626	47	23	70	0.000	285	99	471	0.004
Mental health care	4	0	0	0	0.507	-2	-3	-1	0.000	0	0	0	0.152	-1	-2	0	0.098
Emergency room visits	45,263	-179	-311	-47	0.010	-24,039	-29,236	-18,841	0.000	3	-465	471	0.990	-22,083	-27,629	-16,537	0.000
Inpatient admissions	4,701	-11	-23	1	0.070	-2,813	-3,511	-2,115	0.000	251	163	338	0.000	-1,276	-1,648	-904	0.000
Cervical cancer screening	5,718	-41	-96	14	0.141	-3,854	-4,920	-2,789	0.000	32	-43	107	0.387	-2,320	-3,541	-1,098	0.000
Breast cancer screening	2,076	8	-15	30	0.492	-1,422	-1,908	-937	0.000	-91	-132	-50	0.000	-1,076	-1,617	-534	0.000

The pre-pandemic period is January 1, 2019, to March 31, 2020 (15 months). The pandemic impact period is April 1 to September 30, 2020 (6 months) and the potential resumption period is October 1 to December 31, 2020 (3 months). IMSS, Instituto Mexicano del Seguro Social.

Supplementary table 16

Nepal	Number of districts:	77			Pandemic impact								Potential resumption period			
**Health service**	**Average over the pre-Covid period**	**Avg monthly change before the pandemic (secular trend $\beta1$)**				**Level change due to COVID-19 (change in level $\beta2$)**				**Avg monthly change during the pandemic (change in slope $\beta3$)**				**Remaining level change at the end of 2020 (remaining change in level $\beta4$)**		
		Coeff	LCL	UCL	p-value	Coeff	LCL	UCL	p-value	Coeff	LCL	UCL	p-value	Coeff	LCL	UCL	p-value
Family planning	7,148	-10	-31	11	0.329	-301	-537	-66	0.013	52	-34	139	0.231	154	-277	585	0.479
Antenatal care visits	721	-3	-6	0	0.038	-151	-247	-54	0.003	27	10	44	0.002	-67	-141	7	0.075
Deliveries	418	3	1	5	0.002	-49	-78	-19	0.002	-8	-19	3	0.156	-92	-168	-16	0.018
Caesarean sections	87	1	0	2	0.008	-10	-19	-1	0.033	-1	-5	2	0.398	-29	-51	-7	0.011
Postnatal care visits	128	3	1	4	0.000	-34	-49	-19	0.000	5	2	8	0.004	1	-23	25	0.914
Diarrhea	400	-2	-3	0	0.023	-200	-242	-158	0.000	12	6	17	0.000	8	-16	32	0.525
Pneumonia	139	1	0	2	0.007	-58	-72	-44	0.000	-10	-12	-8	0.000	-108	-130	-87	0.000
BCG	631	2	-2	5	0.356	-288	-368	-209	0.000	49	35	63	0.000	33	-30	96	0.306
Pentavalent	575	-2	-5	0	0.085	-284	-345	-222	0.000	69	55	83	0.000	64	13	114	0.014
Measles	1,035	1	-2	4	0.433	-508	-628	-387	0.000	111	86	136	0.000	103	30	175	0.006
Pneumococcal	530	0	-2	1	0.685	-231	-284	-179	0.000	53	42	65	0.000	42	7	76	0.019
Outpatient visits	17,478	236	149	323	0.000	-3,115	-4,567	-1,664	0.000	-129	-332	73	0.206	-3,915	-5,574	-2,255	0.000
Emergency room visits	2,228	21	7	35	0.004	-180	-555	194	0.341	-227	-332	-122	0.000	-1,107	-1,632	-581	0.000
Inpatient admissions	496	14	-6	33	0.168	-194	-380	-7	0.042	-27	-63	8	0.128	-301	-613	12	0.059
Diabetes visits	317	10	4	16	0.001	-183	-284	-81	0.001	-1	-9	7	0.781	-188	-302	-74	0.002
Hypertension visits	654	23	13	33	0.000	-302	-431	-173	0.000	0	-18	18	0.997	-340	-531	-149	0.001
TB detection	19	0	0	0	0.003	-13	-17	-8	0.000	1	1	2	0.000	-4	-6	-2	0.001
HIV tests	937	30	3	58	0.032	-589	-1,033	-145	0.010	-34	-119	52	0.434	-665	-1,390	59	0.071

In Nepal, the pre-pandemic period is Magh 2075 to Falgun 2077 (January 15, 2019, to March 13, 2020) (14 months). The pandemic impact period is Chaitra 2076 to Bhadra 2077 (March 14, 2020, to September 16, 2020) (6 months) and the potential resumption period is Ashwin to Poush 2077 (September 17, 2020, to January 13, 2021) (4 months).

Supplementary table 17

South Africa		Number of subnational units	11		Pandemic impact								Potential resumption period			
**Health service**	**Average over the pre-Covid period**	**Avg monthly change before the pandemic (secular trend $\beta1$)**				**Level change due to COVID-19 (change in level $\beta2$)**				**Avg monthly change during the pandemic (change in slope $\beta3$)**				**Remaining level change in the last quarter of 2020 (remaining change in level $\beta4$)**			
		Coeff	LCL	UCL	p-value	Coeff	LCL	UCL	p-value	Coeff	LCL	UCL	p-value	Coeff	LCL	UCL	p-value
Antenatal care visits	1,735	-15	-28	-1	0.039	-44	-233	145	0.610	58	7	110	0.031	113	34	192	0.010
Deliveries	1,079	4	-1	8	0.091	-223	-390	-55	0.015	35	20	50	0.001	-123	-244	-2	0.047
Caesarean sections	542	6	-1	12	0.086	-69	-130	-9	0.028	6	3	9	0.002	-76	-160	8	0.070
Postnatal care visits	1,225	7	3	12	0.007	-244	-445	-44	0.022	35	16	54	0.002	-153	-336	31	0.092
Diarrhea	70	3	2	4	0.000	-69	-98	-40	0.000	-1	-4	2	0.413	-55	-75	-34	0.000
Pneumonia	276	-13	-25	-1	0.039	-133	-267	0	0.050	13	4	21	0.011	54	-19	128	0.130
People on ART	130,890	730	197	1,263	0.013	162	-604	928	0.645	-932	-1,989	126	0.077	-6,163	-13,242	916	0.080
Outpatient visits	38,486	-192	-484	99	0.169	-11,955	-25,039	1,128	0.069	235	-364	835	0.398	-7,985	-15,330	-639	0.036
Inpatient admissions	5,338	1	-8	10	0.842	-1,441	-2,712	-169	0.031	101	10	191	0.033	-653	-1,354	47	0.064
Road traffic accidents	376	1	-1	2	0.559	-299	-561	-36	0.030	38	7	70	0.023	-31	-117	56	0.442
Diabetes visits	8,544	-1	-39	37	0.949	-2,033	-4,334	268	0.077	-163	-344	18	0.072	-2,820	-5,949	309	0.072
Cervical cancer screening	1,326	-15	-25	-6	0.005	-870	-1,415	-324	0.006	-9	-37	18	0.453	-694	-1,253	-136	0.020
TB screening	173,332	-431	-1,250	387	0.264	-44,585	-84,801	-4,369	0.033	1,777	-1,658	5,212	0.272	-26,518	-44,268	-8,768	0.008
TB detection	309	-2	-6	2	0.247	-85	-174	3	0.057	1	-8	10	0.841	-36	-66	-6	0.024
TB treatment	309	-3	-8	2	0.251	-77	-153	0	0.049	-1	-11	9	0.770	-39	-75	-4	0.032
Fully vaccinated by 1	1,800	7	4	10	0.001	-420	-789	-52	0.030	47	-5	99	0.071	-103	-172	-35	0.008
BCG vaccine	1,485	6	-2	13	0.115	-380	-715	-46	0.030	35	17	54	0.002	-302	-544	-61	0.020
Pentavalent vaccine	1,609	-1	-5	3	0.673	-240	-606	126	0.172	49	-24	121	0.161	-22	-79	34	0.389
Measles vaccine	1,633	2	-3	8	0.405	-441	-769	-112	0.014	77	21	133	0.013	-62	-181	58	0.273
Pneumococcal vaccine	1,803	5	3	7	0.000	-388	-728	-48	0.030	47	-1	95	0.053	-56	-118	6	0.073
Rotavirus vaccine	1,576	-2	-6	3	0.455	-232	-576	113	0.163	51	-18	120	0.132	13	-41	68	0.593
Trauma admissions	234	1	-2	4	0.562	-121	-231	-10	0.035	14	6	22	0.003	-26	-121	68	0.541

Data are from the Province of KwaZulu-Natal only. The pre-pandemic period is January 1, 2019, to March 31, 2020 (15 months). The pandemic impact period is April 1 to September 30, 2020 (6 months) and the potential resumption period is October 1 to December 31, 2020 (3 months).

Supplementary table 18

South Korea	Number of cities and provinces:	17				Pandemic impact								Potential resumption period			
**Health service**	**Average over the pre-Covid period**	**Avg monthly change before the pandemic (secular trend $\beta 1$)**				**Level change due to COVID-19 (change in level $\beta 2$)**				**Avg monthly change during the pandemic (change in slope $\beta 3$)**				**Remaining level change in the last quarter of 2020 (remaining change in level $\beta 4$)**			
		Coeff	LCL	UCL	p-value	Coeff	LCL	UCL	p-value	Coeff	LCL	UCL	p-value	Coeff	LCL	UCL	p-value
Antenatal care visits	20,811	-150	-242	-57	0.004	-1,271	-2,118	-425	0.006	397	96	698	0.013	654	-7	1,316	0.052
Deliveries	675	-12	-19	-5	0.003	-3	-23	17	0.725	12	3	21	0.010	6	-8	21	0.365
Caesarean sections	738	-6	-10	-2	0.004	-1	-18	16	0.890	11	3	18	0.007	-7	-21	7	0.320
Diarrhea	30,325	-762	-1,267	-258	0.006	-8,011	-13,755	-2,268	0.009	-365	-937	207	0.193	-4,778	-9,646	90	0.054
Pneumonia	10,154	103	41	166	0.003	-6,197	-8,779	-3,614	0.000	-920	-1,393	-447	0.001	-11,730	-17,093	-6,367	0.000
Diabetes visits	173,645	459	143	775	0.007	-5,030	-7,207	-2,853	0.000	1,743	930	2,557	0.000	1,082	-390	2,554	0.138
Hypertension visits	305,043	841	307	1,375	0.004	-10,604	-15,805	-5,403	0.001	3,325	1,564	5,085	0.001	928	-1,325	3,182	0.394
People on ART	386	-1	-2	1	0.270	-12	-22	-1	0.032	3	-1	7	0.146	2	-10	14	0.750
Mental health care	151,528	503	48	958	0.033	-3,937	-5,953	-1,921	0.001	1,439	839	2,039	0.000	627	-1,163	2,418	0.467
Outpatient visits	3,610,980	-21,426	-32,156	-10,696	0.001	-338,782	-553,147	-124,417	0.004	28,937	15,567	42,307	0.000	-192,199	-349,180	-35,217	0.020
Emergency room visits	47,872	-416	-581	-252	0.000	-3,516	-5,519	-1,513	0.002	19	-574	611	0.947	-3,387	-6,266	-508	0.024
Inpatient admissions	82,910	-237	-367	-107	0.001	-3,163	-4,733	-1,593	0.001	-76	-603	452	0.765	-2,731	-4,877	-584	0.016

The pre-pandemic period is January 1, 2019, to March 31, 2020 (15 months). The pandemic impact period is April 1 to September 30, 2020 (6 months) and the potential resumption period is October 1 to December 31, 2020 (3 months).

Supplementary table 19

Thailand	Number of provinces:	77				Pandemic impact								Potential resumption period			
**Health service**	**Average over the pre-Covid period**	**Avg monthly change before the pandemic (secular trend $\beta 1$)**				**Level change due to COVID-19 (change in level $\beta 2$)**				**Avg monthly change during the pandemic (change in slope $\beta 3$)**				**Remaining level change in the last quarter of 2020 (remaining change in level $\beta 4$)**			
		Coeff	LCL	UCL	p-value	Coeff	LCL	UCL	p-value	Coeff	LCL	UCL	p-value	Coeff	LCL	UCL	p-value
Deliveries	332	0	-2	3	0.731	19	6	32	0.006	-2	-7	4	0.574	-21	-54	13	0.226
Hypertension visits	820	5	2	7	0.001	-200	-236	-164	0.000	12	6	18	0.000	153	108	198	0.000
Diabetes visits	358	2	1	3	0.000	-84	-102	-67	0.000	8	4	12	0.000	45	27	64	0.000
Outpatient visits	337,409	1,411	618	2,203	0.001	-109,125	-128,725	-89,525	0.000	8,075	5,788	10,363	0.000	-74,196	-92,632	-55,759	0.000
Inpatient admissions	7,976	-4	-17	10	0.586	-2,483	-2,884	-2,082	0.000	342	260	425	0.000	-815	-1,117	-513	0.000
Road traffic accidents	671	-1	-5	2	0.531	-272	-324	-220	0.000	54	43	65	0.000	68	15	122	0.013
Malaria visits	4	0	0	0	0.002	0	-1	0	0.376	0	-1	0	0.044	0	-1	2	0.359

The pre-pandemic period is January 1, 2019, to March 31, 2020 (15 months). The pandemic impact period is April 1 to September 30, 2020 (6 months) and the potential resumption period is October 1 to December 31, 2020 (3 months).

Supplementary table 20. Population and birth estimates used for calculations of missed health care visits per 1,000

Country	Population [a]	Crude Birth Rate [b]	Estimated Births
Chile	19,166,209	12.19	233,579
Ethiopia	114,963,583	31.90	3,666,878
Ghana	31,072,945	28.99	900,867
Haiti	11,402,533	23.96	273,193
KZN, South Africa	11,531,628[c]	20.13	232,120
Lao PDR	7,275,556	23.12	168,189
Mexico	128,932,753	17.30	2,230,150
Nepal	29,136,808	19.58	570,440
South Korea	51,780,579	5.90	305,505
Thailand	69,799,978	10.17	709,726

[a] The World Bank, World Development Indicators (2020). Population, total - Chile, Ethiopia, Ghana, Haiti, Lao PDR, Mexico, Nepal, Korea, Rep., Thailand. Retrieved from https://data.worldbank.org/indicator/SP.POP.TOTL?locations=CL-ET-GH-HT-LA-MX-NP-KR-TH

[b] The World Bank, World Development Indicators (2020). Birth rate, crude (per 1,000 people) - Chile, Ethiopia, Ghana, Haiti, Lao PDR, Mexico, Nepal, Korea, Rep., Thailand. Retrieved from https://data.worldbank.org/indicator/SP.DYN.CBRT.IN?locations=CL-ET-GH-HT-LA-MX-NP-KR-TH

[c] Statistics South Africa. (2020). (rep.). Statistical release: Mid-year population estimates. Retrieved from http://www.statssa.gov.za/publications/P0302/P03022020.pdf
