# Supplementary material for: COVID-19 and resilience of healthcare systems in ten countries
Source: Nat Med. 2022 Mar 14;28(6):1314–24. doi: 10.1038/s41591-022-01750-1 (PMC9205770; doi:10.1038/s41591-022-01750-1)
Supplement: Supplementary file 2 — Reporting Summary [file 41591_2022_1750_MOESM2_ESM.pdf]

nature portfolio

Corresponding author(s): Catherine Arsenault

Last updated by author(s): February 14, 2022

Reporting Summary

Nature Portfolio wishes to improve the reproducibility of the work that we publish. This form provides structure for consistency and transparency in reporting. For further information on Nature Portfolio policies, see our Editorial Policies and the Editorial Policy Checklist.

Statistics

For all statistical analyses, confirm that the following items are present in the figure legend, table legend, main text, or Methods section.

n/a	Confirmed	
☐	☒	The exact sample size (*n*) for each experimental group/condition, given as a discrete number and unit of measurement
☐	☒	A statement on whether measurements were taken from distinct samples or whether the same sample was measured repeatedly
☐	☒	The statistical test(s) used AND whether they are one- or two-sided<br>*Only common tests should be described solely by name; describe more complex techniques in the Methods section.*
☐	☒	A description of all covariates tested
☐	☒	A description of any assumptions or corrections, such as tests of normality and adjustment for multiple comparisons
☐	☒	A full description of the statistical parameters including central tendency (e.g. means) or other basic estimates (e.g. regression coefficient) AND variation (e.g. standard deviation) or associated estimates of uncertainty (e.g. confidence intervals)
☐	☒	For null hypothesis testing, the test statistic (e.g. *F*, *t*, *r*) with confidence intervals, effect sizes, degrees of freedom and *P* value noted<br>*Give P values as exact values whenever suitable.*
☒	☐	For Bayesian analysis, information on the choice of priors and Markov chain Monte Carlo settings
☒	☐	For hierarchical and complex designs, identification of the appropriate level for tests and full reporting of outcomes
☐	☒	Estimates of effect sizes (e.g. Cohen's *d*, Pearson's *r*), indicating how they were calculated

Our web collection on statistics for biologists contains articles on many of the points above.

Software and code

Policy information about availability of computer code

Data collection	In six countries (Ethiopia, Ghana, Haiti, KwaZulu-Natal in South Africa, Lao PDR and Nepal), RHIS data were extracted from the DHIS2 platform. https://dhis2.org/
Data analysis	All analyses were conducted using STATA version 16. All code supporting this analysis can be accessed from: https://github.com/catherine-arsenault/HS-performance-during-covid-do-files/tree/master/Multi-country/Paper%201%20Service%20utilization

For manuscripts utilizing custom algorithms or software that are central to the research but not yet described in published literature, software must be made available to editors and reviewers. We strongly encourage code deposition in a community repository (e.g. GitHub). See the Nature Portfolio guidelines for submitting code & software for further information.

Data

Policy information about availability of data

All manuscripts must include a data availability statement. This statement should provide the following information, where applicable:

- Accession codes, unique identifiers, or web links for publicly available datasets
- A description of any restrictions on data availability
- For clinical datasets or third party data, please ensure that the statement adheres to our policy

The data used in this study were collected from multiple sources. In Chile, data are publicly available from https://deis.minsal.cl/. The data from the Mexican Institute for Social Security were deposited in a repository: https://dataverse.harvard.edu/dataset.xhtml?persistentId=doi:10.7910/DVN/XSHQYB. In Thailand, data are publicly available from the Ministry of Public Health: http://hdcservice.moph.go.th/. In all other countries, the data are restricted, and permissions to access the data must be obtained from Ministries of Health.

Field-specific reporting

Please select the one below that is the best fit for your research. If you are not sure, read the appropriate sections before making your selection.

☐ Life sciences ☒ Behavioural & social sciences ☐ Ecological, evolutionary & environmental sciences

For a reference copy of the document with all sections, see nature.com/documents/nr-reporting-summary-flat.pdf

Behavioural & social sciences study design

All studies must disclose on these points even when the disclosure is negative.

Study description	We performed a quantitative observational study using a prospective single interrupted time-series design with data before and after the declaration of the pandemic.
Research sample	This research was undertaken as part of the QuEST network, a new initiative focused on increasing the impact and scale of research on health systems through multi-country partnerships. As per the QuEST approach, we aimed for representation of countries from different regions and with different income levels, health system types, and severity of COVID-19. The initial outreach was to researchers and policy makers with past collaborations on health system research. Authors initially invited potential collaborators from 18 countries; 10 were able to assemble the required data. The resulting research sample includes health facilities located in 10 countries (Chile, Ethiopia, Ghana, Haiti, Laos, Mexico, Nepal, South Africa, South Korea and Thailand). In four countries (Ghana, Haiti, Nepal, South Korea), these data represent all health facilities in the country. In Ethiopia, the data represent all health facilities in the country except for those in the Tigray region which was excluded due to the ongoing conflict. In Lao PDR, Mexico, and Thailand the data included only public sector facilities that provide services to 50%-70% of the population. It was not possible to obtain data from private sector facilities in these countries. In Chile, certain indicators were available from both public and private hospitals (deliveries, c-sections, inpatient admissions, hospital discharges for child pneumonia, and road traffic accidents) while other indicators included only public sector facilities. In South Africa, the data were from all facilities in the KwaZulu-Natal province and included public and private sectors in the province.
Sampling strategy	The data were not sampled; they represent the full set of health facilities that report to these data systems in every country (or region in the case of KwaZulu-Natal).
Data collection	The data extraction was conducted by local researchers or Ministry of Health staff with permissions to access the administrative or routine health information systems (RHIS) data sources.
Timing	Data extraction began in April 2020 and ended in August 2021. Important delays often occur for these data systems to be updated. We included data from January 2019 to December 2020 in the main analysis. We also included data for a subset of indicators and countries until May or June 2021 in supplemental materials.
Data exclusions	In Ethiopia, the Tigray region was excluded because health facilities stopped reporting to the DHIS2 in November 2020 due to the ongoing conflict. We conducted thorough data cleaning in the six health systems with facility- or municipality-level information (Chile, Ethiopia, Haiti, KwaZulu-Natal, Nepal, and Laos). In these countries, we included only health facilities that reported each indicator for at least 15 months out of 24. This led to stable numbers of facilities reporting each month and allowed us to assess the effect of the pandemic more accurately. Thus, a small number of health facilities with sparse reporting was excluded. The number of health care visits excluded varies according to the indicator and country and is described in the methods.
Non-participation	The analysis uses administrative and RHIS data from health facilities. Non-participation is not applicable.
Randomization	There was no randomization as this is an observational study.

Reporting for specific materials, systems and methods

We require information from authors about some types of materials, experimental systems and methods used in many studies. Here, indicate whether each material, system or method listed is relevant to your study. If you are not sure if a list item applies to your research, read the appropriate section before selecting a response.

Materials & experimental systems

n/a	Involved in the study	
☒	☐	Antibodies
☒	☐	Eukaryotic cell lines
☒	☐	Palaeontology and archaeology
☒	☐	Animals and other organisms
☒	☐	Human research participants
☒	☐	Clinical data
☒	☐	Dual use research of concern

Methods

n/a	Involved in the study	
☒	☐	ChIP-seq
☒	☐	Flow cytometry
☒	☐	MRI-based neuroimaging
